# Partial least squares path modeling using ordinal categorical indicators

**DOI:** 10.1007/s11135-016-0401-7

**Published:** 2016-09-14

**Authors:** Florian Schuberth, Jörg Henseler, Theo K. Dijkstra

**Affiliations:** 1 Faculty of Business Management and Economics, University of Würzburg, Sanderring 2, 97070 Würzburg, Germany; 2 Faculty of Engineering Technology, University of Twente, P.O. Box 217, 7500 AE Enschede, The Netherlands; 3 Faculty of Economics and Business, University of Groningen, Nettelbosje 2, P.O. Box 800, 9747 AE Groningen, The Netherlands

**Keywords:** Structural equation models, Consistent partial least squares, Ordinal categorical indicators, Common factors, Composites, Polychoric correlation

## Abstract

This article introduces a new consistent variance-based estimator called ordinal consistent partial least squares (OrdPLSc). OrdPLSc completes the family of variance-based estimators consisting of PLS, PLSc, and OrdPLS and permits to estimate structural equation models of composites and common factors if some or all indicators are measured on an ordinal categorical scale. A Monte Carlo simulation (N $=500$) with different population models shows that OrdPLSc provides almost unbiased estimates. If all constructs are modeled as common factors, OrdPLSc yields estimates close to those of its covariance-based counterpart, WLSMV, but is less efficient. If some constructs are modeled as composites, OrdPLSc is virtually without competition.

**Electronic supplementary material:**

The online version of this article (doi:10.1007/s11135-016-0401-7) contains supplementary material, which is available to authorized users.

## Introduction

Structural equation modeling (SEM) has become an established method in the fields of business and social sciences. Its capacity to model constructs, to take into account various forms of measurement error, and to test entire theories makes it a prime candidate for encountering a variety of research issues.

For SEM two types of estimators need to be differentiated: covariance- and variance-based estimators. Covariance-based parameter estimates are obtained by minimizing the distance between the empirical variance-covariance matrix of the indicators and its theoretical counterpart. Variance-based estimators, on the contrary, create proxies for constructs first and subsequently estimate model parameters based on theses proxies. While covariance-based methods are preferred if the model contains constructs modeled as common factors, variance-based estimators are favoured if the underlying model consists of constructs modeled as composites, in particular, when the composites are endogenous in the structural model.

Among variance-based estimators, partial least squares (PLS) path modeling is regarded as the “most fully developed and general system” (McDonald 1996, p. 240) and it was even called a “silver bullet” (Hair et al. 2011). The use of PLS path modeling is prevalent in many fields, e.g., information systems research (Marcoulides and Saunders 2006) or marketing (Hair et al. 2012). Because of its capability to model both factors and composites,^1^ the latest version of PLS, known as consistent PLS, is a vigorous method for estimation and is acknowledged by researchers across different disciplines. Common factors can be used to model constructs of behavioral research such as attitudes or personality traits, whereas composites can be applied to model strong concepts (Höök and Löwgren 2012), i.e., the abstraction of artefacts such as management instruments, innovations, or information systems. Consequently, PLS path modeling is a preferred statistical tool for success factor studies (Albers 2010).

Recently, a lot of development has been done in the field of PLS path modeling. For example, a new criterion for discriminant validity based on heterotrait-monotrait ratio of common factor correlations (Henseler et al. 2015), the standardized root mean square residual (SRMR) as a measure of overall model fit (Henseler et al. 2014), and bootstrap-based tests for overall model fit (Dijkstra and Henseler 2015a) were introduced. Since PLS creates composites as proxies for all kinds of constructs, its estimates suffer from attenuation and are biased in case of an underlying common factor model (Schneeweiss 1993). Therefore, a consistent PLS (PLSc) version was developed which corrects for this bias to consistently estimate SEMs with common factors (Dijkstra and Henseler 2015b). All these developments are based on the PLS algorithm and therefore on ordinary least squares (OLS) regression analysis implicitly assuming that all indicators are continuous.

Since numerous studies are based on data collected by questionnaires, the indicators used are rarely measured on a metric scale. Hence, in many situations researchers are faced with data measured on ordinal categorical scales, e.g., in marketing research, in particular customer satisfaction surveys (Hair et al. 2012; Coelho and Esteves 2007).

It is well known in the PLS path modeling literature as well as in other fields that treating categorical variables as continuous can lead to biased estimates and therefore to invalid inferences and erroneous conclusions. Lohmöller recognizes that the “$\left[\ldots\right]$ standard procedures cannot be used for the categorical and ordinal-scaled variables $\left[ ...\right]$” (Lohmöller 2013, Chap. 4). Also Hair et al. (2012) mention that PLS is often used with categorical indicators but that their use in a procedure like PLS which uses OLS as estimator can be problematic. Several approaches to address this issue in the context of PLS are provided by the literature, e.g., ordinal PLS (OrdPLS) an innovative approach to deal with ordinal categorical indicators in a psychometric way (Cantaluppi 2012; Cantaluppi and Boari 2016). As OrdPLS is based on the traditional PLS algorithm, its use is limited to models where all constructs are modeled as composites. However, researchers often deal with models containing constructs modeled as common factors instead of composites (Ringle et al. 2012; Hair et al. 2012). So, there is a real need for improving methods like OrdPLS to be able to deal with common factors, composites and ordinal categorical indicators.

**Fig. 1:**
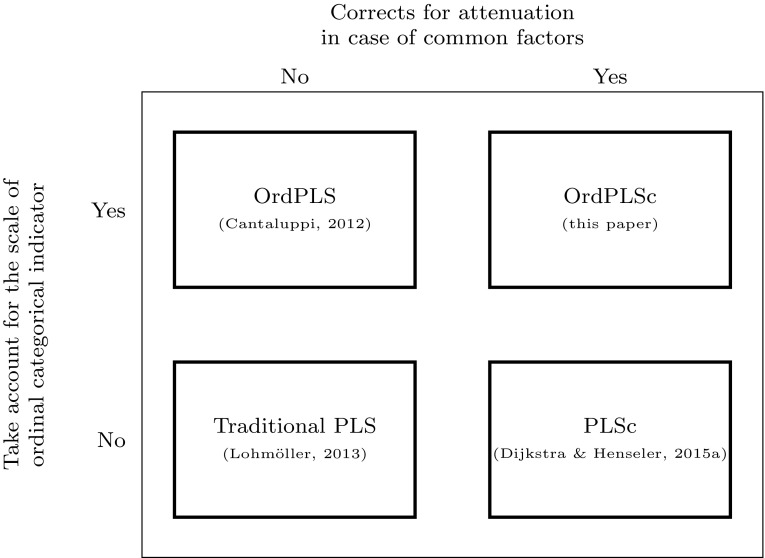
A typology of PLS methods

We provide such a development and contribute to the literature an extension of OrdPLS called ordinal consistent partial least squares (OrdPLSc). It combines the advantages of both, OrdPLS and PLSc. Hence, OrdPLSc is an estimator which enables researchers to consistently estimate structural equation models including not only composites, but common factors and ordinal categorical indicators too. Figure 1 contrasts the properties of traditional PLS, PLSc, OrdPLS, and OrdPLSc with respect to dealing with common factors and taking into account the scale of ordinal categorical indicators.

We run a Monte Carlo simulation to investigate the performance of OrdPLSc in different conditions and compare it as benchmark to means and variance adjusted weighted least squares (WLSMV). The latter approach is a consistent covariance-based estimator which is typically used for structural equation models with common factors in case of ordinal categorical indicators. Moreover, we show how traditional PLS, PLSc, and OrdPLS behave for different kinds of models and show how PLS and PLSc are affected when the scale of ordinal categorical indicators is ignored.

The remainder of the paper is organized as follows: The next section shows the development from PLS to PLSc and provides a reformulation of these two procedures in terms of indicators correlation matrices. In Sect. 3 we give a literature review of existing approaches dealing with categorical indicators in the framework of PLS, in particular we present the idea of the OrdPLS approach. In Sect. 4 we introduce ordinal consistent PLS (OrdPLSc) to the literature. In the following Sect. 5, we present the design of our Monte Carlo simulation, which is conducted to examine the performance of OrdPLSc and different other estimators under several conditions. We present these findings in Sect. 6. The article closes with the discussion of the results in Sect. 7. An Appendix covers the figure of the threshold parameter distribution.

## The development from PLS path modeling to consistent PLS path modeling

PLS was developed by Wold (1975) for the analysis of high dimensional data in a low-structure environment and has undergone various extensions and modifications. It is an approach similar to generalized canonical correlation analysis (GCCA), and in addition able to emulate several of Kettenring (1971)’s techniques for the canonical analysis of several sets of variables (Tenenhaus et al. 2005).

In its most modern appearance known as consistent PLS (PLSc) (Dijkstra and Henseler 2015a, b), it can be understood as a well-developed SEM method. It is capable to estimate recursive and non-recursive structural models with constructs modeled as composites and common factors. Both obtain the outer weights and the final stand-ins for the constructs by the classical PLS algorithm. While traditional PLS simply relies on OLS to estimate the model parameters, its extended version, PLSc, uses two-stage least squares (2SLS) to consistently estimate even non-recursive path models. Furthermore, PLSc is able to handle both constructs modeled as composites and as common factors by using a post-correction for attenuation for correlations between common factors, and common factors and indicators.

**Fig. 2:**
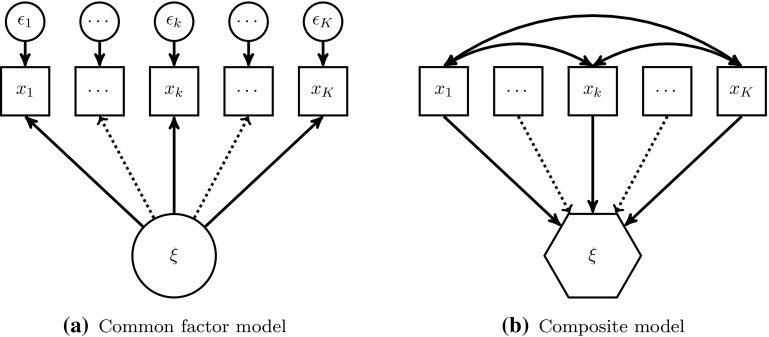
Common factor versus composite

The classical common factor model assumes that the variance of a block of indicators $(x_{1},\ldots ,x_{K})$ is completely explained by the underlying common factor ($\xi$ in the large circle) and by their random errors $(\epsilon _{1},\ldots ,\epsilon _{K})$, see Fig. 2a. Hence, the indicators reflect the underlying common factor (reflective measurement model). This sort of indicator is also known as effect indicators (Bollen and Bauldry 2011). Common factors are usually used in behavioral research.

As Fig. 2b depicts, composites ($\xi$ in the hexagon) are formed as linear combinations of their belonging indicators $(x_{1},\ldots ,x_{K})$. Since the indicators form the composite, they are related to composite-formative measurement models.^2^ Furthermore, the composite model does not put any restrictions on the covariances of the indicators belonging to one block, hence, it relaxes the assumption that all covariation between the indicators has to be explained by the common factor. Composites are often used as proxies for scientific concepts of interest (Ketterlinus et al. 1989; Maraun and Halpin 2008; Tenenhaus 2008; Rigdon 2012).

For the derivation of OrdPLS(c) it is crucial to describe the well-known PLS algorithm (Wold 1975) and its extension to PLSc in terms of indicator covariances or correlations, respectively. Since in PLS no distinction between exogenous and endogenous constructs is made, we use the following notation: ${\varvec{\eta }}$ is a $(J\times 1)$ vector containing all modeled constructs which are connected by the structural model, whether they are modeled as common factors or as composites. The $(K \times 1)$ vector ${\varvec{x}}$ contains all indicators which measure the common factors or build the composites, respectively.

### Partial least squares

For a sample of size *n*, all observations of the *K* indicators are stacked in a data matrix ${\varvec{X}}$ of dimension $(n\times K)$. For simplicity, the $K_j$ indicators belonging to one common factor or one composite $\eta _j$ are grouped to form block *j* with $j=1,\ldots ,J$. Observations of block *j* are stacked in the data matrix ${\varvec{X}}_j$ of dimension $(n\times K_j)$ with $\sum \nolimits _{j=1}^{J}{K_j}=K$. Furthermore, we assume that each indicator is standardized, as is customary in PLS, to have mean zero and unit variance, such that the indicators’ sample covariance matrix ${\varvec{S}}$ equals the sample correlation matrix.

The PLS estimation procedure consists of three parts. In the first part, for each block *j* initial arbitrary outer weights $\hat{{\varvec{w}}}_{j}^{(0)}\,(K_j\times 1)$ are chosen which satisfy the following condition: $\hat{{\varvec{w}}}_j^{(0)\prime } {\varvec{S}}_{jj} \hat{{\varvec{w}}}_j^{(0)}=1$ where the $K_j\times K_j$ matrix ${\varvec{S}}_{jj}$ contains the sample correlations of the indicators of block *j*. This condition holds for all outer weights in each iteration *i* and can be achieved by using the scaling factor $(\hat{{\varvec{w}}}_j^{(i)\prime }{\varvec{S}}_{jj} \hat{{\varvec{w}}}_j^{(i)})^{-\frac{1}{2}}$ for the outer weights $\hat{{\varvec{w}}}_{j}^{(i)}$ in each iteration.

In the second part, the iterative PLS algorithm starts with step one, the outer approximation of $\eta _j$:

1 $$\begin{aligned} \hat{{\varvec{\eta }}}_j^{(i)}={\varvec{X}}_j \hat{{\varvec{w}}}_j^{(i)} \quad {\text {with}}\quad \hat{{\varvec{w}}}_j^{(i)\prime }{\varvec{S}}_{jj} \hat{{\varvec{w}}}_j^{(i)}= 1, \end{aligned}$$

where $\hat{{\varvec{\eta }}}_j^{(i)}$ is a column vector of length *n*. Since outer weights are scaled, all outer proxies also have mean zero and unit variance.

In the second step, the inner proxy of $\eta _j$ is calculated as a linear combination of inner weights and outer proxies of $\eta _{j'}$:

2 $$\begin{aligned} \tilde{{\varvec{\eta }}}_j^{(i)}=\sum \limits _{j'=1}^{J}{e_{jj'}^{(i)}\hat{{\varvec{\eta }}}_{j'}^{(i)}}, \end{aligned}$$

where $\tilde{{\varvec{\eta }}}_j^{(i)}$ is again a column vector of length *n*. The inner weight $e_{jj'}$ defines how the inner proxy $\tilde{{\varvec{\eta }}}_j$ is built. Three different schemes for the calculation of $e_{jj'}$ are commonly used: *centroid* (Wold 1982), *factorial* (Lohmöller 2013), and *path weighting*. However, all schemes yield essentially the same results (Noonan and Wold 1982), hence, we only consider the *centroid* scheme.^3^ The inner weights are chosen according to the signs of the correlations between the outer proxies

3 $$\begin{aligned} e_{jj'}^{(i)}= {\left\{ \begin{array}{ll} {\text {sign}}(\hat{{\varvec{w}}}_j^{(i)\prime }{\varvec{S}}_{jj'}\hat{{\varvec{w}}}_{j'}^{(i)}), \quad &{}{{\text {for}}\, j\ne j' \, \text {and if construct}}\, j \,{\mathrm{and}}\, j' \, {\mathrm{are\, adjacent}} \\ 0, \quad &{}{\text {otherwise}}, \end{array}\right. } \end{aligned}$$

where adjacent refers to the constructs *j* and $j'$ directly connected by the structural model. All inner proxies $\tilde{{\varvec{\eta }}}_j^{(i)}$ are again scaled to have unit variance.

In the third and last step of the iterative part, new outer weights are calculated. This can be done in three ways: *mode A*, *mode B*, and *mode C*. For *mode A*, estimated outer weights of block *j* equal the estimated coefficients of a multivariate regression from the indicators of block *j* on its related inner proxy. Due to standardization, the new estimated outer weights $\hat{{\varvec{w}}}_j^{(i+1)}$ equal the correlations between the inner proxy and its related indicators:

4 $$\begin{aligned} \hat{{\varvec{w}}}_j^{(i+1)}\propto \sum \limits _{j' = 1}^{J}{{\varvec{S}}_{jj'} \hat{{\varvec{w}}}_{j'}^{(i)} e_{jj'}^{(i)}} \quad {\text {with}} \quad \hat{{\varvec{w}}}_j^{(i+1)\prime }{\varvec{S}}_{jj} \hat{{\varvec{w}}}_j^{(i+1)} = 1. \end{aligned}$$

In contrast, for *mode B*, the new outer weights equal the estimated coefficients of a regression from the inner proxy on its connected indicators:

5 $$\begin{aligned} \hat{{\varvec{w}}}_j^{(i+1)}\propto {\varvec{S}}_{jj}^{-1} \sum \limits _{j'=1}^{J}{{\varvec{S}}_{jj'}\hat{{\varvec{w}}}_{j'}^{(i)} e_{jj'}^{(i)}} \quad {\text {with}}\quad \hat{{\varvec{w}}}_j^{(i+1)\prime }{\varvec{S}}_{jj} \hat{{\varvec{w}}}_j^{(i+1)} = 1. \end{aligned}$$

*Mode C*, also known as *MIMIC mode*, is a mixture of *mode A* and *mode B* and is not considered here.^4^

As the traditional PLS algorithm has no single optimization criteria to be minimized, the new outer weights $\hat{{\varvec{w}}}_j^{(i+1)}$ are checked for significant changes compared to the outer weights from the previous iteration step $\hat{{\varvec{w}}}_j^{(i)}$. If there is a significant change in the weights, the algorithm starts again at step one by building new outer proxies with the new outer weights, otherwise it stops.

In the last part, the obtained stable outer weights $\hat{{\varvec{w}}}_j$ are used to build final composite stand-ins for both common factors and composites: $\hat{{\varvec{\eta }}}_j={\varvec{X}}_j\hat{{\varvec{w}}}_j$. For constructs which are modeled as common factors, the factor loadings are estimated by OLS in accordance with the measurement model. In contrast, for constructs which are modeled as composites the final weights equal the stable weights from the last iteration. Finally, path coefficients are estimated by OLS with respect to the structural model.

### Consistent PLS

PLS is based on composites, which implies that estimates are biased if constructs are modeled as common factors.^5^ In general, a composite model has larger absolute inter composite correlations compared to the absolute inter common factor correlations of a model with the same structure but where all constructs are modeled as common factors. However, a transformation of the model-implied correlation matrix of a composite model into the model-implied correlation matrix of a common factor model can be achieved by a correction for attenuation (Cohen et al. 2013, Chap. 2.10). Consistent PLS (PLSc) uses this correction to obtain consistent estimates for models containing common factors (Dijkstra and Henseler 2015a, b). The correction requires that each common factor is measured by at least two indicators and uses the proportionality between the population outer weights and the population factor loadings, ${\varvec{w}}_j= c_j {\varvec{\lambda }}_j$. The estimated correction factor for block *j* satisfies the following condition

6 $$\begin{aligned} {\text {plim}}(\hat{c}_j)=\sqrt{{\varvec{\lambda }}_j^\prime {\varvec{\Sigma }}_{jj} {\varvec{\lambda }}_j}, \end{aligned}$$

where ${\varvec{\lambda }}_j$ is a column vector of length $K_j$ containing the population loadings of common factor $\eta _j$ and ${\varvec{\Sigma }}_{jj}$ is the $K_j\times K_j$ population correlation matrix of the indicators of block *j*.^6^ The correction factor $\hat{c}_j$ can be obtained as

7 $$\begin{aligned} \hat{c}_j^2=\frac{\hat{{\varvec{w}}}_j'({\varvec{S}}_{jj}-{\text {diag}}({\varvec{S}}_{jj}))\hat{{\varvec{w}}}_j}{\hat{{\varvec{w}}}_j'(\hat{{\varvec{w}}}_j \hat{{\varvec{w}}}_j'-{\text {diag}}(\hat{{\varvec{w}}}_j \hat{{\varvec{w}}}_j'))\hat{{\varvec{w}}}_j}. \end{aligned}$$

It is chosen such that the Euclidean distance between

8 $$\begin{aligned} {\varvec{S}}_{jj} -{\text {diag}}({\varvec{S}}_{jj}) \quad {\text {and}}\quad (c_j\hat{{\varvec{w}}}_j)(c_j \hat{{\varvec{w}}}_j)'-{\text {diag}}((c_j\hat{{\varvec{w}}}_j)(c_j \hat{{\varvec{w}}}_j')) \end{aligned}$$

is minimized (Dijkstra and Henseler 2015a). Factor loadings of block *j* are consistently estimated by

9 $$\begin{aligned} \hat{{\varvec{\lambda }}}_j=\hat{c}_j \hat{{\varvec{w}}}_j. \end{aligned}$$

Moreover, PLSc is able to consistently estimate the path coefficients of recursive and non-recursive models^7^ using OLS or 2SLS according to the structural model. Since all variables are standardized, the estimated path coefficients are based on the correlations between the columns of $\hat{{\varvec{\eta }}}\,(n \times J)$. The correlation between the common factors *j* and $j'$ is consistently estimated by:

10 $$\begin{aligned} \widehat{\text {cor}}(\eta _j,\eta _{j'})=\frac{\hat{{\varvec{w}}}_j'{\varvec{S}}_{jj'} \hat{{\varvec{w}}}_{j'}}{\hat{c}_j(\hat{{\varvec{w}}}_j'\hat{{\varvec{w}}}_j)\hat{c}_{j'}(\hat{{\varvec{w}}}_{j'}' \hat{{\varvec{w}}}_{j'})}. \end{aligned}$$

Using the corrected correlation of Eq. (10) for the estimation of the structural model, one obtains consistently estimated path coefficients between the common factors.^8^ For constructs which are modeled as composites no correction of the correlation is required because, by construction, they are not affected by attenuation. In case construct *j* is modeled as a common factor and construct $j'$ as a composite, the consistently estimated correlation is obtained as

11 $$\begin{aligned} \widehat{{{\mathrm{\text {cor}}}}}(\eta _j,\eta _{j'})=\frac{\hat{{\varvec{w}}}_j'{\varvec{S}}_{jj'} \hat{{\varvec{w}}}_{j'}}{\hat{c}_j(\hat{{\varvec{w}}}_j'\hat{{\varvec{w}}}_j)}. \end{aligned}$$

## The development from PLS to ordinal PLS

Since incorrectly handling ordinal categorical variables as continuous can lead to biased inferences and therefore to erroneous conclusions, the literature provides several approaches to deal with discrete indicators: dichotomize the ordinal categorical indicator, a mixture of PLS and correspondence analysis (CA), Partial Maximum Likelihood PLS (PML-PLS), and non-metric PLS (NM-PLS).

Common practice in PLS is to replace a categorical indicator by a dummy matrix which is known as dichotomizing. Since each categorical indicator is replaced by $s-1$ dummy variables, where *s* is the number of observed categories, $s-1$ outer weights are obtained for the original variable. This contradicts the idea of treating an indicator as a whole.

Betzin and Henseler (2005) use correspondence analysis to quantify ex-ante categorical indicators. As the quantified indicators are obtained, PLS is used to estimate the model parameters. As a result, individual weights are obtained for each category of the categorical indicator. Again, this has the drawback that no single outer weight for a categorical indicator is calculated.

Partial Maximum Likelihood Partial Least Squares (PML-PLS) (Jakobowicz and Derquenne 2007) is a modified version of the original PLS algorithm. It is a combination of PLS and generalized linear models designed to deal with indicators of any scale. For categorical indicators, individual outer weights are computed for each category by ANOVA. Based on those, one ’global’ weight per categorical indicator is calculated. However, statistical properties like the proportionality of outer weights to factor loadings are unknown for the global weights and further investigation is needed. Moreover, the authors note that PML-PLS “is especially advantageous in the case of nominal or binary variables” (Jakobowicz and Derquenne 2007) but we focus on ordinal categorical indicators.

The last approach, non-metric partial least squares (NM-PLS) extends PLS by an alternating least squares optimal scaling (ALSOS) algorithm to quantify qualitative indicators and gain outer weights (Russolillo 2012). ALSOS is a procedure which quantifies qualitative variables by preserving properties of the original measurement scales and optimizes an objective optimization criteria by alternating least squares (Young 1981). In the case of NM-PLS, the categorical indicator is quantified in a way that the correlation between the inner proxy and the quantified categorical indicator is maximized. As a result for each indicator one outer weight is obtained as in traditional PLS for continuous indicators.

However, the evaluation of the presented approaches is based on empirical studies and, to our knowledge, no simulation studies have been conducted to investigate their statistical properties. For an extension to PLSc in order to deal with common factors, it is necessary that the outer weights are proportional to the factor loadings. Moreover, the modified PLS procedures are often applied to common factor models which represents a misspecified model in the context of PLS. Hence, an assessment of their statistical properties is hardly possible and we decided not to pursue any of the previously mentioned methods.

### Ordinal PLS

A promising approach to deal with ordinal categorical indicators is ordinal PLS (OrdPLS^9^) (Cantaluppi 2012). It is a modified procedure for handling ordinal categorical variables in a classical psychometric way. In Sect. 2 we showed that all parameters can be obtained by the use of the correlation matrix ${\varvec{S}}$. Traditional PLS uses the Bravais-Pearson (BP) correlation matrix which requires all indicators to be continuous for consistency. The observation of an ordinal categorical variable is a qualitative measure, yet it is often coded as numeric and therefore mistakenly treated as quantitative by researchers. This routinely happens in applications with binary and ordinal categorical indicators which results in biased BP correlation estimates (Quiroga 1992; O’Brien and Homer 1987; Wylie 1976; Carroll 1961). To fix this, OrdPLS uses a consistent correlation matrix as input for the traditional PLS algorithm. An advantage of OrdPLS over the approaches previously introduced is its transparent way of dealing with ordinal categorical variables. Moreover, the original PLS algorithm remains untouched and it is just provided by a consistent correlation matrix as input for the algorithm.

Since OrdPLS does not correct for attenuation, it shows the same drawbacks as PLS if common factors are included in the model. Nevertheless, we consider OrdPLS as a powerful extension of PLS when applied under appropriate circumstances, i.e., for models with only composites. Furthermore, it is straightforward to extend by PLSc, to overcome its drawback for common factor models, see Sect. 4. In the following subsection we present Pearson’s considerations of ordinal categorical variables to provide a better understanding of the polychoric and polyserial correlation.

### Ordinal categorical variables according to Pearson

Pearson (1900, 1913) considers an ordinal categorical variable as a crude measure of an underlying continuous random variable, while Yule (1900) assumes categorical variables being inherently discrete. In this paper we follow Pearson’s idea: an observed ordinal categorical indicator *x* is the result of a polytomized standard normally distributed random variable $x^*$:

12 $$\begin{aligned} x=x_{i} \quad {\text {if}} \quad \tau _{i-1}\le x^*< \tau _{i} \quad i=1,\ldots ,s \end{aligned}$$

where the threshold parameters $\tau _0, \ldots , \tau _s$ determine the observed categories. The first and last threshold are fixed: $\tau _{0}=-\infty$ and $\tau _{s}=\infty$. Moreover, thresholds are assumed to be strictly increasing: $\tau _{0}<\tau _{1}<\ldots <\tau _s$.

**Fig. 3:**
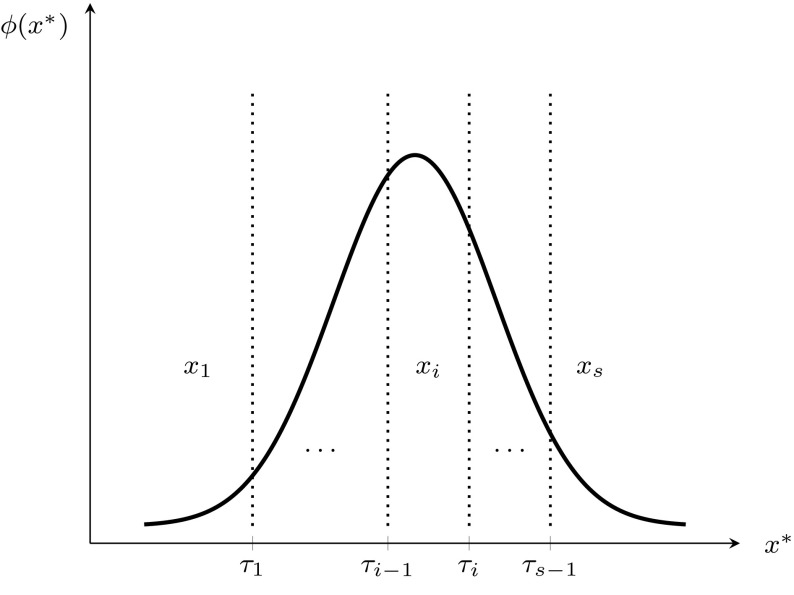
Pearson’s idea of an ordinal categorical variable

Figure 3 depicts the idea of an underlying continuous variable: For indicator *x* category $x_{i}$ is observed if the realisation of the underlying continuous variable $x^*$ is in between $\tau _{i-1}$ and $\tau _{i}$.

### Polychoric and polyserial correlation

Since an ordinal categorical variable is determined by an underlying continuous variable, it is more appropriate to consider the correlation between these underlying quantitative continuous variables for evaluating the linear relationship of interest. This is achieved by using the polychoric or the polyserial correlation (Drasgow 1988). To present the principles of the polychoric correlation, we consider two ordinal categorical variables $x_1$ and $x_2$ with consecutive categories $i=1,\ldots ,s$ and $j=1,\ldots ,r$. They are constructed in the way presented in Eq. (12). The two underlying continuous variables $x_1^*$ and $x_2^*$ are assumed to be jointly bivariate standard normally distributed with correlation $\rho$. The correlation between $x_1^*$ and $x_2^*$ can be estimated by maximum likelihood using the following log-likelihood function:

13 $$\begin{aligned} \ln L=\ln (c)+\sum \limits _{i=1}^{s}{\sum \limits _{j=1}^{r}{n_{ij}\ln (\pi _{ij})}}, \end{aligned}$$

where $\ln (c)$ is a constant term, $n_{ij}$ denotes the observed joint absolute frequency of $x_1=i$ and $x_2=j$, and $\pi _{ij}$ is the probability that category *i* and *j* are observed jointly. Due to the joint normality assumption, $\pi _{ij}$ is obtained as:

14 $$\pi _{ij}=\Phi _2(\tau _{x_{1i}},\tau _{x_{2j}},\rho )-\Phi _2(\tau _{x_{1i}},\tau _{x_{2j-1}},\rho )-\Phi _2(\tau _{x_{1i-1}},\tau _{x_{2j}},\rho )+\Phi _2(\tau _{x_{1i-1}},\tau _{x_{2j-1}},\rho ),$$

where $\Phi _2$ is the cumulative distribution function of the bivariate standard normal distribution. The parameters $\tau _{x_{1i}}$, $\tau _{x_{2j}}$, and $\rho$ are chosen to maximize the function $\ln L$. In order to reduce computational burden, a two-step procedure can be used (Olsson 1979). In the first step, threshold parameters are estimated separately as quantiles of cumulative marginal frequencies, i.e., $\hat{\tau }_{x_{1i}}=\Phi ^{-1}(p_i)$ where $p_i$ equals the cumulative marginal relative frequency up to category *i* and the function $\Phi ^{-1}$ represents the quantile function of the standard normal distribution (analogous for $x_2$). Second, given the estimated threshold parameters, Eq. (13) is maximized with respect to $\rho$. In case of a continuous and an ordinal categorical variable, the correlation between the two continuous variables is obtained by the polyserial correlation (Olsson et al. 1982). For more than two variables, a multivariate version is used to estimate the correlations (Poon and Lee 1987). Moreover, a less computational intensive two-step approach can be used for the multivariate version (Lee and Poon 1987). OrdPLS as well as OrdPLSc makes use of the polychoric and polyserial correlation when ordinal categorical indicators are part of the model.

## Ordinal consistent partial least squares

**Fig. 4:**
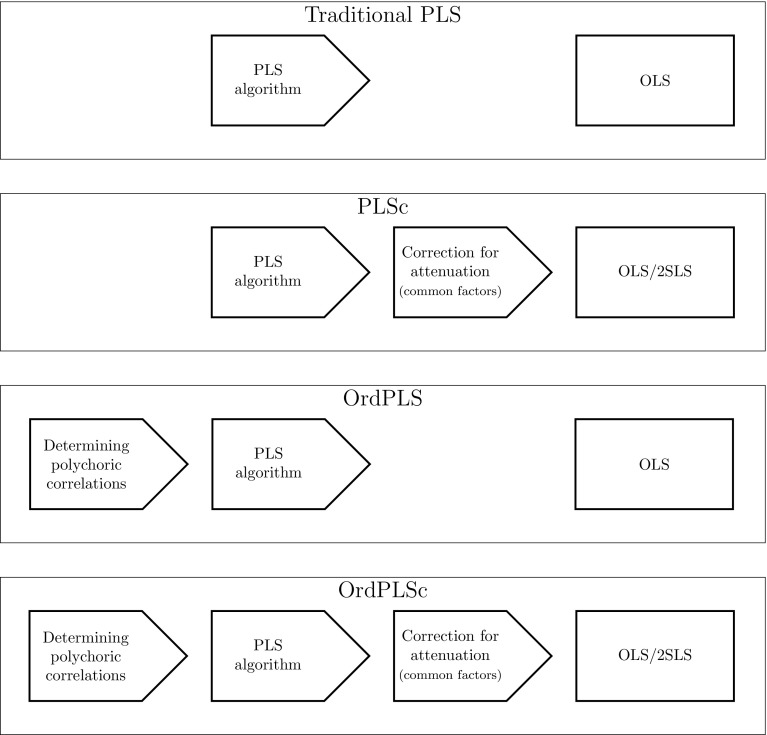
Conceptual differences between the four PLS approaches

We introduce a new approach which deals with common factors, composites, and ordinal categorical indicators. It is called ordinal consistent partial least squares (OrdPLSc) and is a combination of OrdPLS and PLSc. It uses the polychoric correlation, a consistent correlation matrix in case of ordinal categorical indicators, as input for the PLS algorithm and corrects for attenuation if common factors are included in the model. Since the use of the polychoric correlation matrix does not affect the original PLS algorithm, the proportionality property of the outer weights is maintained and the correction of attenuation can be applied to the inter-composite correlation matrix in the same manner as in PLSc. Figure 4 illustrates commonalities and differences of the three previously presented PLS approaches and OrdPLSc.

**Fig. 5:**
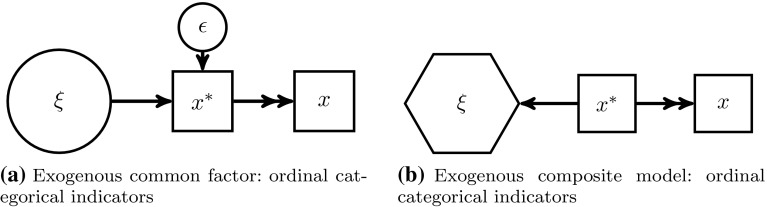
Ordinal categorical indicators in common factor and composite models

The role of an ordinal categorical indicator *x*, more precisely its underlying continuous variable $x^*$, is influenced by its position in the model. As Fig. 5a displays, when the ordinal categorical indicator belongs to a common factor, its outcome is indirectly influenced by the underlying common factor and a measurement error $\epsilon$ through the underlying continuous variable $x^*$. An ordinal categorical indicator that is part of a composite, see Fig. 5b, is simply a crude measure of an underlying continuous variable (represented by a double headed arrow) which actually builds the composite along with other indicators belonging to this block.

To ignore the nature of the ordinal categorical indicators may cause serious problems. First, in common factor models the correlation between the indicator and its underlying factor is underestimated (Quiroga 1992; O’Brien and Homer 1987), which leads to biased estimates. Second, in the case of a composite, disregarding the scale of the ordinal categorical indicator leads to biased estimates, too. This is well known as the *error-in-variables problem* (see, e.g., Wooldridge 2012, Chap. 15).

## Monte Carlo simulation

In order to investigate the performance of OrdPLSc under various conditions and to compare it with PLSc, OrdPLS, and PLS for structural equation models containing ordinal categorical indicators, we ran a Monte Carlo simulation. In particular, we considered their unbiasedness and their efficiency, the most important properties of an estimator. Furthermore, we studied the bias of PLS and OrdPLS estimates for common factor models with ordinal categorical indicators. Also for PLSc, which is known to be a consistent estimator in the framework of continuous indicators (Dijkstra and Henseler 2015a), we examined the behavior when ordinal categorical variables are used instead of continuous ones.

We conducted a Monte Carlo simulation with 1000 multivariate standard normally distributed samples with 500 observations each. The continuous indicators were categorized in the way presented in Sect. 3.2. We only considered consecutive categories, i.e., $1,2,\ldots ,s$. To compare all estimators in a fair way, inadmissible solutions^10^ were removed and replaced by proper estimations before evaluation.

We considered the following experimental conditions: two population models (a model with three common factors and a model with one common factor and two composites), four different numbers of categories (2, 3, 5, and, 7 categories), and five different distributions of the ordinal categorical indicators (symmetric, moderate asymmetric, extreme asymmetric, alternating moderate asymmetric, and alternating extreme asymmetric). Each condition was estimated by OrdPLSc, PLSc, OrdPLS, and PLS. As a benchmark comparison for the pure common factor model we also estimated the model by WLSMV, a consistent covariance-based three stage least squares estimator (Muthén 1984; Lee et al. 1990b), which is considered the golden standard for common factor models with ordinal categorical indicators.^11^

### Two population models

Starting point were two kinds of models: one model with only common factors and one model with one common factor and two composites. The pure common factor model was chosen to compare OrdPLSc to its covariance-based counterpart WLSMV. In designing the path structure of the models, we chose a structure used several times in the literature (Hwang et al. 2010; Henseler 2012; Henseler and Sarstedt 2013).

#### Population model with only common factors

**Fig. 6:**
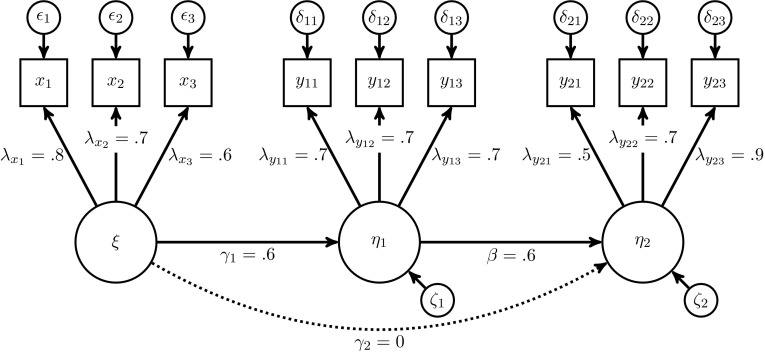
Population model with three common factors

First we considered a pure common factor model with the following population structural equations

15 $$\begin{aligned}&\eta _1=\gamma _1 \xi _1 + \zeta _1\end{aligned}$$

16 $$\begin{aligned}&\eta _2=\gamma _2 \xi _1 + \beta _{21} \eta _1 + \zeta _2, \end{aligned}$$

where $\gamma _1=0.6, \gamma _2=0.0, \beta _{21}=0.6, {{\mathrm{\text {var}}}}(\zeta _1)=0.64 , {{\mathrm{\text {var}}}}(\zeta _2)=0.64$, and ${{\mathrm{\text {cov}}}}(\zeta _1, \zeta _2)=0$. As Fig. 6 depicts, each common factor was reflectively measured by three indicators with factor loadings 0.8, 0.7, 0.6 for $\xi$, 0.7, 0.7, 0.7 for $\eta _1$, and 0.5, 0.7, 0.9 for $\eta _2$.

All measurement errors and structural residuals were mutually independent as well as all common factors were assumed to be independent of the measurement errors. Therefore, the indicators population correlation matrix is given by:17


#### Population model with two composites and one common factor

**Fig. 7:**
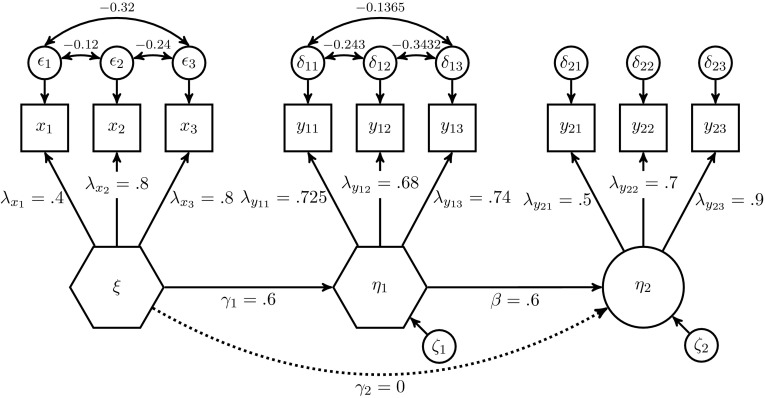
Population model with two composites and one common factor

Second, we considered a model with the identical structural model used for the model with three common factors, but two of the constructs were modeled as composites instead of common factors. Figure 7 depicts the population model in terms of common and composite factors. We deliberately chose this representation of the composites and not the one used in Fig. 2 to clarify the construction of the population correlation matrix of the indicators.

Here $\xi$ and $\eta _1$ are constructs modeled as composites. Since the relationship between a composite and its indicators can be expressed by composite loadings (Fig. 7) or weights, we also reported the weights: the composites were formed by their connected indicators: $\xi ={\varvec{x}}' {\varvec{w}}_{{\varvec{x}}}$ where ${\varvec{w}}_{{\varvec{x}}}^\prime =(0.3, 0.5, 0.6)$ and $\eta _1={\varvec{y}_{1}}^\prime {\varvec{w}}_{{\varvec{y}_{1}}}$ where ${\varvec{w}}_{{\varvec{y}_{1}}}^\prime =(0.4, 0.5, 0.5)$. The common factor $\eta _2$ was again measured by three indicators with the following loadings: 0.5, 0.7, and 0.9.

The population correlation matrix of the indicators has the following form:18


### Number of categories

We considered four different numbers of indicator categories: 2, 3, 5, and 7. An increasing number of categories diminishes the bias of the BP correlation (O’Brien and Homer 1987). Hence, we expect a decreasing difference between PLS and OrdPLS as well as PLSc and OrdPLSc as the number of categories increases.

### Threshold parameter distribution

We investigated differently skewed ordinal categorical indicators by varying threshold parameter distributions for each number of categories. We considered threshold distributions used in the literature before (Rhemtulla et al. 2012): symmetric, moderately asymmetric, extremely asymmetric, alternating moderately asymmetric, and alternating extremely asymmetric distributed threshold parameters. In the alternating asymmetric threshold distribution scenario, the same thresholds were used, but the direction of asymmetry was reversed for the indicators $x_2$, $y_{11}$, $y_{13}$, and $y_{22}$.^12^ Since BP correlations are more downward biased for more asymmetrical threshold distributions (Bollen and Barb 1981; Faber 1988; Holgado-Tello et al. 2010) and even more for alternating skewed indicators (Olsson 1980), we expect an increasing difference between OrdPLSc and PLSc estimates as well as OrdPLS and PLS estimates from the symmetrical to the alternating extreme threshold distribution.

### Data generation and analysis

All simulations were conducted within the R (version 3.2.2) statistical programming environment (R Core Team 2015). Multivariate standard normally distributed data sets were drawn using the *mvrnorm* function of the *MASS* package (Venables and Ripley 2002). To obtain PLS and PLSc estimates, we primarily used functions provided by the *matrixpls* package (Rönkkö 2015), which allows the use of the empirical correlation matrix as input for PLS and PLSc. A slightly modified version of those functions was also used for OrdPLS and OrdPLSc. The modified version is provided by the authors upon request. Since *matrixpls* is still under development we also partly verified our results obtained with ADANCO (Henseler and Dijkstra 2015). The polychoric correlation was calculated by the *polychoric* function from the *psych* package (Revelle 2015) using the two-step approach.^13^ WLSMV estimation was carried out using the *lavaan* package (Rosseel 2012).

## Results

This section shows the results of our study.^14^ In the following, we summarize our findings in terms of bias with respect to the quality of the parameter estimates for the model containing only common factors and the mixed model. The bias is the deviation of the estimated parameter mean across all Monte Carlo simulation runs from its population counterpart

19 $$\begin{aligned} {\text {Bias}}=\frac{1}{1000}\sum \limits _{i=1}^{1000}{\hat{\theta }_i}-\theta \end{aligned}$$

where $\theta$ represents the population parameter and $\hat{\theta }$ is the estimated parameter. The bias statistic provides information about the estimators’ unbiasedness and is used as one performance measure to compare OrdPLSc estimates with estimates from approaches commonly applied. Moreover, we assessed the estimators’ efficiency in terms of average standard deviation across all Monte Carlo simulation runs. We finish by summarizing inadmissible results, i.e., Heywood cases.

In general, for the moderate asymmetric and the alternating moderate asymmetric threshold parameter distribution the estimators led to similar results. The same was observed for extremely and alternating extremely distributed thresholds. For latter conditions, all estimators showed a poorer performance, which confirmed our expectations.

### Bias of the parameter estimates

**Fig. 8:**
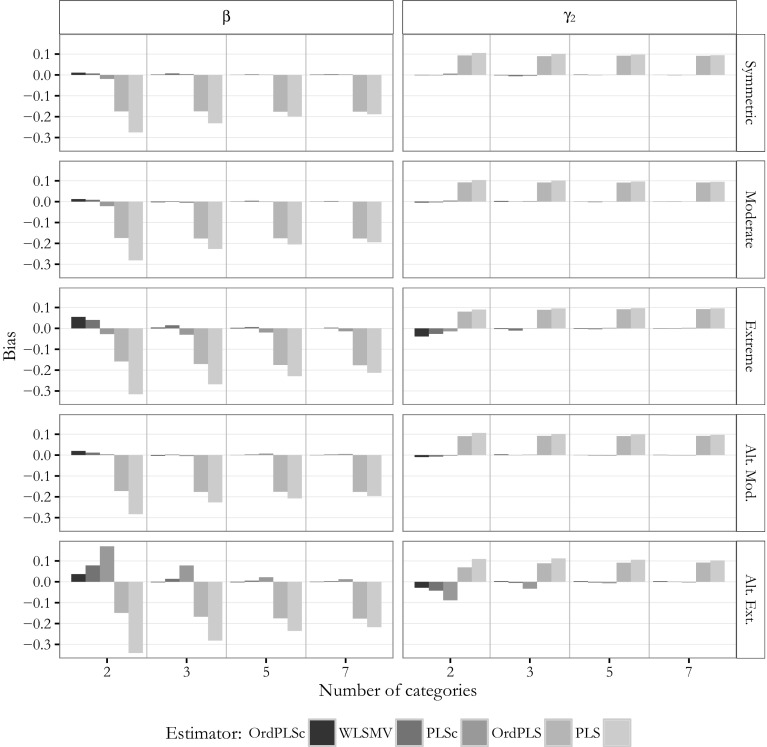
Model with only common factors: bias for $\beta$ and $\gamma _2$

**Fig. 9:**
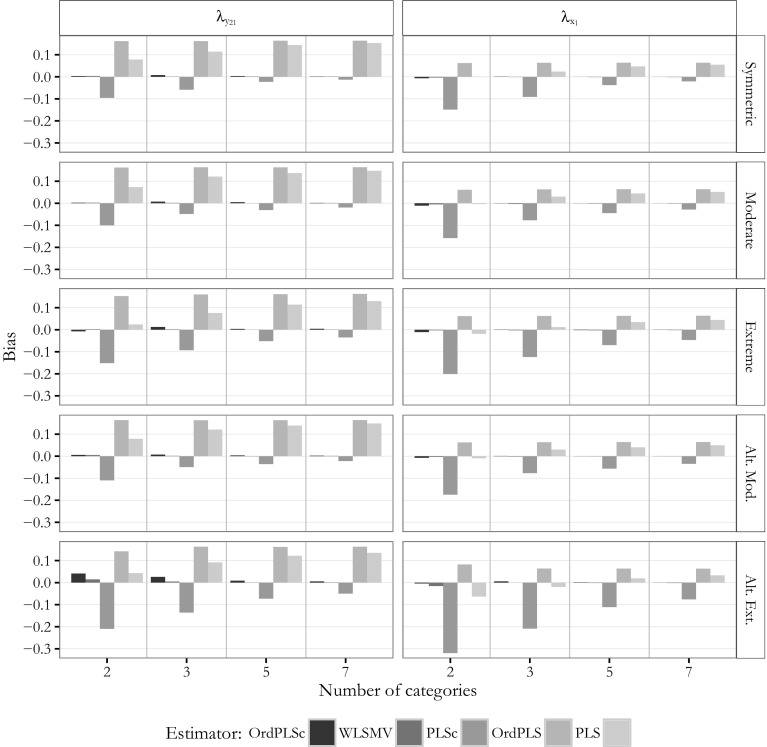
Model with only common factors: bias for $\lambda _{y_{21}}$ and $\lambda _{x_1}$

Figures 8 and 9 display the bias of the path coefficient estimates for $\beta$(=0.6) and $\gamma _2$(=0.0), and factor loading estimates for $\lambda _{x_1}$(=0.5) and $\lambda _{y_{21}}$(=0.8) of the pure common factor model for the different number of categories and the different threshold parameter distributions. Due to space constraints, we omit the results for the estimated path coefficient $\hat{\gamma }_1$ and the other factor loading estimates. They behaved very similar to the ones presented.

These figures make clear that OrdPLSc and WLSMV led to almost the same results for the estimated path coefficients and factor loadings under all conditions. Both estimators were hardly biased. Only in case of extremely and alternating extremely skewed indicators slightly biased estimates were obtained. This bias diminished with an increasing number of categories.

In contrast, PLSc path coefficient estimates behaved surprisingly well in most of the conditions. The estimated path coefficients were biased for extremely asymmetrically distributed threshold parameters and even more biased for the alternating extreme threshold parameter distribution. The population zero-path $\gamma _2$ was approximately unbiased in almost every condition except for alternating extremely distributed threshold parameters with 2 categories. This bias declined with an increase in the number of categories. In contrast, factor loading estimates were downward-biased in all conditions but the bias dramatically declined as the number of categories increased. However, the bias was still present for 7 categories.

We obtained different results for OrdPLS which led to a fairly constant bias in all conditions unaffected by the number of categories. In particular, the estimated path coefficients $\hat{\beta }$ and $\hat{\gamma }_2$ were downward-biased while the estimated zero-path coefficient $\hat{\gamma }_1$ was upward-biased. Factor loading estimates were all upward biased, except for the estimates of the largest factor loading $\lambda _{y_{23}}=0.9$ which were only slightly biased. This bias was largely unaffected by the number of categories.

PLS produced the most biased path coefficient estimates for $\gamma _1$ and $\beta _1$. While the bias of OrdPLS was fairly constant in all conditions, the bias of the PLS estimates converged to the bias of the OrdPLS estimates with an increasing number of categories. A similar pattern was observed for PLS factor loading estimates. For 2 categories, factor loading estimates were slightly biased, but the bias became more pronounced and converged to the bias of the OrdPLS factor loading estimates as the number of categories increased.

**Fig. 10:**
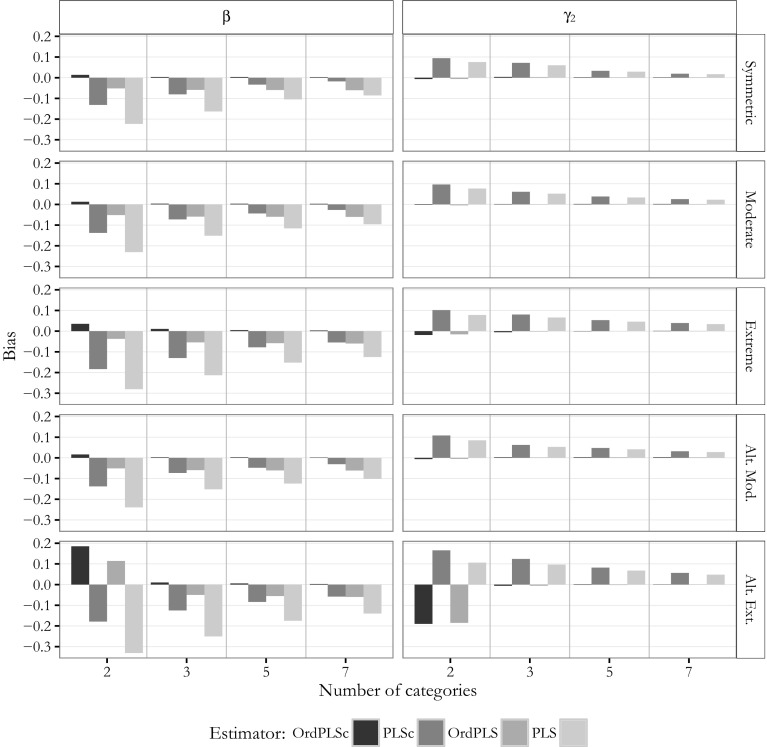
Mixed model: bias for $\beta$ and $\gamma _2$

**Fig. 11:**
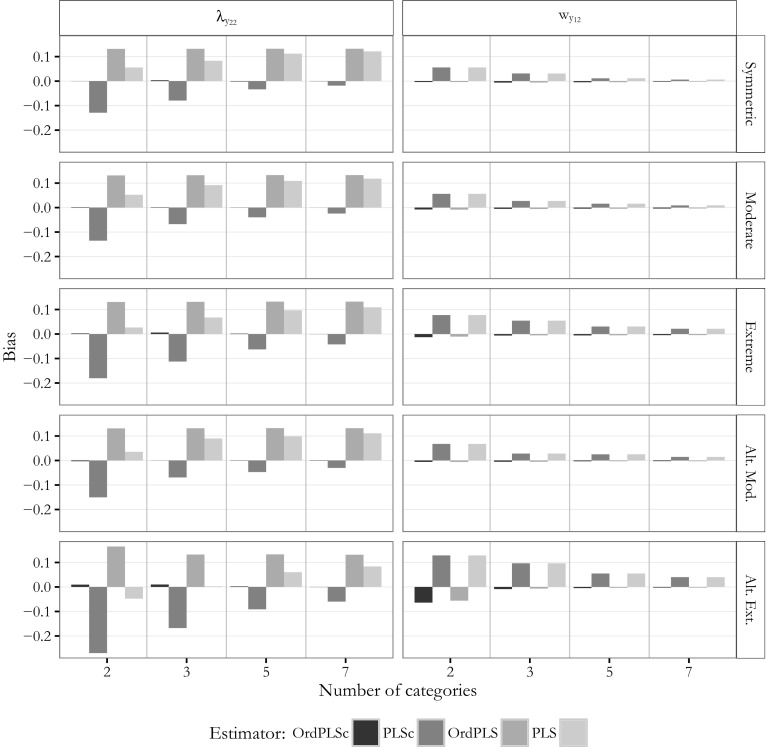
Mixed model: bias for $\lambda _{y{22}}$ and $w_{y_{12}}$

Next, we examined the estimates obtained for the mixed population model. Again, for the sake of simplicity, Figs.  10 and 11 only depict the bias of the estimates for the path coefficients $\beta$(=0.6) and $\gamma _2$(=0), for the factor loading $\lambda _{y_{22}}$(=0.7) and for the weight $w_{y_{12}}$(=0.5) of the model with two composites and one common factor.

The OrdPLSc estimator led to almost unbiased path coefficient, factor loading, and weight estimates under the considered conditions. Only for an alternating extreme asymmetric threshold parameter distribution, path coefficient estimates were clearly biased for two categories. However, this bias disappeared for more than two categories.

OrdPLS led to very similar results compared to OrdPLSc for estimates affected only by composites ($\hat{\gamma }_1$ and weights). The estimated zero-path $\hat{\gamma }_2$ was also unbiased except for alternating extremely skewed indicators with 2 categories, while the path coefficient estimate $\hat{\beta }$ which is only affected by a common factor was constantly biased. Factor loading estimates were again all upward-biased under almost every threshold parameter distributions. This bias was neither affected by the number of categories nor by the threshold parameter distribution.

In contrast, PLSc path coefficient estimates were all biased. This bias was more pronounced by the asymmetry of the threshold parameter distribution. Moreover, factor loadings were underestimated. In general, factor loading estimates showed a very similar behavior as the estimated factor loadings from the model with only common factors. Most weight estimates were only slightly biased, but estimates for $w_{x_2}$ and $w_{y_1}$ showed a clear bias. All biases decreased and PLSc estimates converged to the OrdPLSc estimates as the number of categories increased.

PLS produced almost the same biased estimates for path coefficient $\gamma _1$ and the weights as PLSc. The other path coefficients were also biasedly estimated under all conditions. While this bias decreased with an increasing number of categories, the upward-biased factor loading estimates became even more biased for an increasing number of categories. Again, average PLS factor loading estimates tended to converge to OrdPLS average factor loading estimates.

### Efficiency

Apart from unbiasdness, an estimator’s efficiency is of interest to assess its quality. Therefore, we evaluated the standard deviations of the standardized path coefficient, loading, and weight estimates. In general, all standard deviations decreased with an increasing number of categories, but increased for more asymmetric threshold parameter distributions.

Considering the pure common factor model, WLSMV was always more efficient than OrdPLSc. Since comparing estimators efficiency is only meaningful for unbiased or slightly biased estimates, the other results for the pure common factor model are not evaluated.

Also the estimates for the composite model became more efficient with an increasing number of categories. For estimated parameters between composites only, PLS and PLSc as well as OrdPLS and OrdPLSc produced almost the same standard errors. Estimated parameters connected with at least one common factor showed larger standard deviations for OrdPLSc than OrdPLS. In most cases, path coefficient and weight estimates were less efficient for OrdPLS than PLS, while factor loadings were more efficiently estimated by OrdPLS.

### Inadmissible solutions

**Fig. 12:**
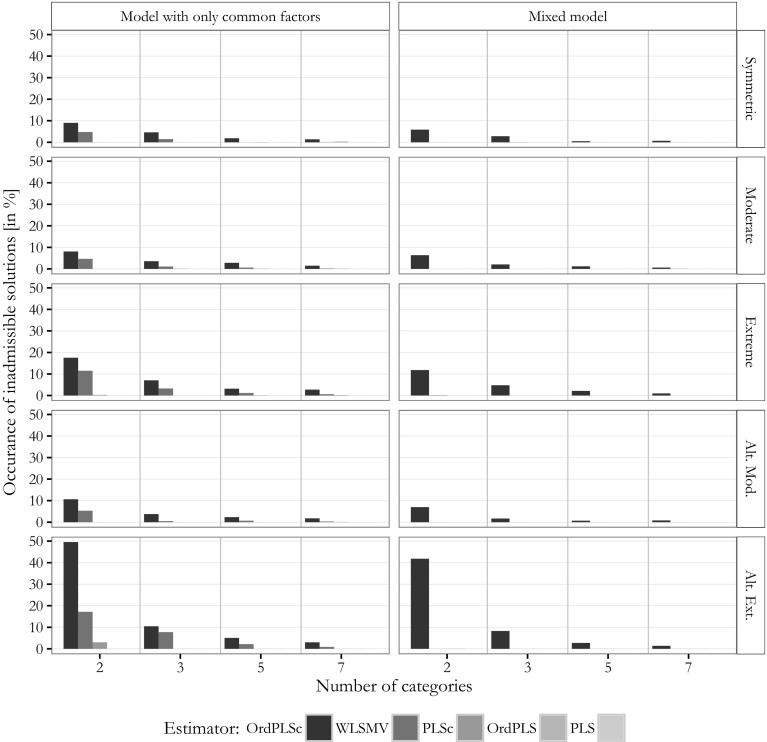
Inadmissible solutions

We finish the results part by comparing the inadmissible solutions. Inadmissible solutions are results with absolute factor loadings greater than one, a non positive semi-definite construct correlation matrix, or results where the estimation algorithm did not converge. Figure 12 depicts the relative frequencies of inadmissible results.

PLS, OrdPLS, and PLSc produced almost no inadmissible solutions for both kind of models. In contrast, OrdPLSc and WLSMV produced a few inadmissible solutions under every condition. The total number of inadmissible results increased for more skewed distributed indicators. The most inadmissible results were produced for alternating extremely distributed threshold parameters.

A similar pattern was observed for inadmissible results during the bootstrap procedure. PLS and OrdPLS again produced no improper solutions. In general, the number of inadmissible results during the bootstrap procedure increased for PLSc with an increasing number of categories, while it decreased for OrdPLSc and WLSMV.

## Discussion

The first goal of our study was to propose a variance-based estimator for structural equation models that is able to consistently estimate models with common factors, composites, and ordinal categorical indicators. We developed OrdPLSc combining the approaches and thus favorable characteristics of OrdPLS and PLSc.

Our results confirmed that OrdPLSc fulfills its intended purpose. For a sample size of 500 observations, OrdPLSc factor loading, weight, as well as path coefficient estimates were almost unbiased under every condition. As the combination of the polychoric correlation and PLSc led to larger standard errors of parameter estimates, OrdPLSc produced a few improper solutions in terms of absolute factor loadings larger than 1. The number of inadmissible solutions was mainly driven by the estimates of the largest factor loading $\lambda _{y_{23}}$. However, the number of inadmissible solutions were in an acceptable range. Compared to WLSMV, OrdPLSc produced very similar estimates but with larger standard errors and a few more inadmissible solutions. However, OrdPLSc outperformed PLS, OrdPLS, and PLSc in terms of bias for both models, which makes OrdPLSc to be the dominant approach under the considered variance-based estimators if ordinal categorical indicators are included in the model. In case of model parameters which are not connected to a common factor, OrdPLSc and OrdPLS as well as PLSc and PLS produced almost the same estimates and standard errors. This is not surprising, as no correction for attenuation is needed, which is the only difference between OrdPLSc and OrdPLS, and PLSc and PLS, respectively.

Second, we investigated the behavior of PLSc, OrdPLS, and PLS in different scenarios using ordinal categorical indicators. Although PLSc uses the BP correlation and therefore does not account for the scale of ordinal categorical indicators, it was surprisingly accurate in estimating the path coefficient of the model with only common factors in most conditions. This could be due to the use of identical threshold parameters for the indicators, but further research is needed.^15^ Furthermore, PLSc behaved as expected, factor loadings were underestimated and the bias increased for more asymmetric threshold parameter distribution, which is due to the downward-bias of the BP correlation. This bias declined as the number of categories increased because the bias of the BP correlation decreased. Therefore, the use of PLSc for models with both common factors and composites is appropriate but only for indicators with a large number of categories. In our simulation study, 7 categories were not enough for the bias to disappear completely.

Moreover, our findings support the results of Cantaluppi (2012) that OrdPLS path coefficient estimates are less biased than PLS estimates in the pure common factor model. Although it takes into account the scale of ordinal categorical indicators, the problem of attenuation remains unaddressed which led to downward-biased estimated path coefficients and upward-biased estimated factor loadings. As this bias was almost unaffected by the number of categories and the indicators’ distribution, OrdPLS estimates were constantly biased. However, OrdPLS accurately estimated the model parameters which were not connected to common factors because no correction for attenuation is needed. Therefore, OrdPLS is an appropriate estimator for models containing only constructs modeled as composites.

Traditional PLS suffers from two shortcomings: no correction for attenuation in case of common factors and not accounting for the scale of ordinal categorical indicators. For a small number of categories the bias of attenuation and the bias of the BP correlation cancelled out, which led to only slightly biased factor loading estimates. When the number of categories increased, the bias of the BP correlation decreased and PLS factor loading estimates became more and more inaccurate and converged to the OrdPLS estimates, which do not suffer from the bias of the BP correlation. Therefore, PLS should be cautiously used for models containing common factors regardless whether ordinal categorical indicators are included or not.

Since OrdPLSc uses the polychoric correlation which assumes normality for the latent variables underlying each ordinal categorical indicator, it cannot be declared anymore as an approach which is free of distributional assumptions. However, the assumption of joint normality of the underlying unobservable variables can be relaxed, as the polychoric correlation produces fairly unbiased correlation estimates for elliptically symmetric distributed variables (Kukuk 1999). Furthermore, due to the nature of the ordinal categorical indicators, point estimates of factor scores or composite scores should not be directly calculated from their observations. To overcome this shortcoming procedures like the *mode estimation*, *median estimation*, or *mean estimation* can be used (Cantaluppi 2012). This issue currently limits the use of OrdPLSc for prediction.^16^

In our simulation study, we only considered situations where all indicators were measured on an ordinal categorical scale. In empirical research practice, continuous indicators are often included in the model. In such a situation, the polyserial or BP correlation should be used, too, to estimate the population correlation matrix. Future research should investigate the behavior of OrdPLSc for models containing a mixture of ordinal categorical and continuous indicators. As a study is limited to its design, we further recommend to investigate the behavior of OrdPLSc, in particular, for small sample sizes. In more general, we recommend to investigate the use of the polychoric correlation in other variance-based estimators which can be expressed in terms of indicators correlation matrix, e.g., generalized structural component analysis (Hwang and Takane 2014).

### Electronic supplementary material

Below is the link to the electronic supplementary material.

Supplementary material 1 (PDF 103 kb)

## Figures and Tables

**Fig. 13:**
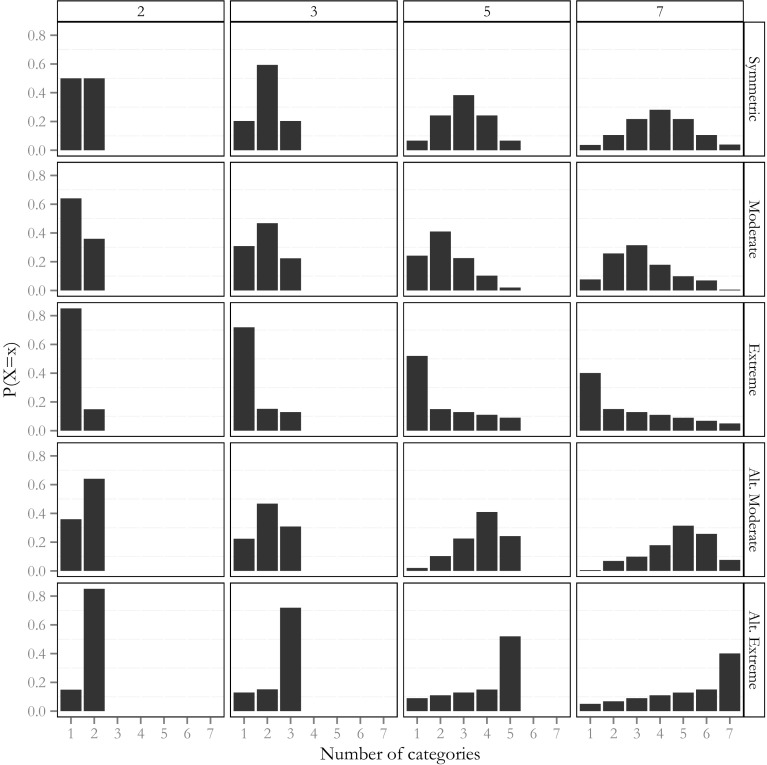
Threshold parameter distributions

## Footnotes

1 For a comparison of constructs modeled as composites or common factors, see Rigdon (2012).

2 In general, the literature provides two definitions of a formative measurement model: (i) the (composite) indicators which completely determine composite (Fornell and Bookstein 1982), and (ii) the (causal) indicators which do not completely explain the underlying latent variable. See Bollen and Bauldry (2011) for a more detailed description.

3 For more details on the other schemes, see Tenenhaus et al. (2005).

4 A consistent version of *mode C*, for any of its 2^*J*^ − 2 implementations, can be obtained by using the properties of *mode A* and *mode B*, see Dijkstra (1981, 1985, Chap. 2, par. 5.2), but since *mode C* is intermediate between the other modes, adding *mode C* does not really contribute to a further understanding.

5 Both, common factors as well as composites are legit ways of construct modeling, see Rigdon (2012).

6 The use of *mode B* for common factors is not considered here. For a consistent version of PLS using *mode B* see Dijkstra (1981, 2011).

7 PLSc relaxes the assumptions of the *basic design* (Wold 1982) where non-recursive structural models are not allowed.

8 For more details, e.g., the consistent estimation of non-recursive models and the correction for nonlinear structural equation models see Dijkstra (1983, 1981, 1985, 2010, 2011), Dijkstra and Schermelleh-Engel (2014).

9 OrdPLS was originally called OPLS (Cantaluppi 2012). An anonymous reviewer suggested to use a different name in order to avoid confounding with O-PLS (Trygg and Wold 2002). We came to an agreement with Cantaluppi to speak of OrdPLS in the future. We thank the anonymous reviewer for suggesting such a disambiguation.

10 Inadmissible solutions are estimations with absolute factor loadings larger than 1, non positive-definite construct correlation matrix, or estimations which have not converged.

11 The mixed model with an endogenous composite cannot be estimated by WLSMV because of identification problems. Moreover, as in OrdPLS and OrdPLSc, covariance-based estimators for categorical indicators are typically based on polychoric correlation, see Lee et al. (1990a, 1992), De Leon (2005), Liu (2007), Katsikatsou et al. (2012).

12 For an exact description of the threshold parameter distribution, see the Appendix.

13 If the polychoric correlation matrix was not positive definite an eigenvector smoothing was done to assure its positive definiteness. Moreover, we followed the recommendation of Savalei (2011) and used the ‘ADD’ approach (0.5) for empty cells in the case of two categories and the ‘NONE’ approach else. The same was done for WLSMV.

14 The complete results are provided in the supplementary material.

15 Since the BP correlation is about to be proportional biased (for a certain range of correlations, see Kukuk 1991), bias cancels out for path coefficients and only affects factor loading and correction factor estimates. Results may change for indicators with a different number of categories and different threshold parameter distribution.

16 This issue is subject of current research by Florian Schuberth and Gabriele Cantaluppi.
