# Supplementary material for: Partial least squares path modeling using ordinal categorical indicators
Source: Qual Quant. 2016 Sep 14;52(1):9–35. doi: 10.1007/s11135-016-0401-7 (PMC5794891; doi:10.1007/s11135-016-0401-7)
Supplement: Supplementary file 1 — Supplementary material 1 (PDF 103 kb) [file 11135_2016_401_MOESM1_ESM.pdf]

Supplementary material: Partial least squares path modeling using ordinal categorical indicators

Quality & Quantity

In the following the results of the ADD approach for two categories and the results of the NONE approach for more than two categories are presented. These are the results used in the paper. Every second row contains the average standard deviations of the parameter estimates. The last four columns contain the number of inadmissible results

- *Load*: absolute factor loading is larger than 1.
- *CompCor*: Correlation matrix between the composites/common factors is non positive-definite.
- *notc*: The PLS algorithm is not converged.
- *Boot*: number of inadmissible solutions during the bootstrap procedure.

Table 1: Results for the model with three common factors

Threshold dist.	Estimator	# cat	γ_1=0.6	$\gamma_2 = 0.0$	$\beta = 0.6$	$\lambda_{x_1} = 0.8$	$\lambda_{x_2} = 0.7$	$\lambda_{x_3} = 0.6$	$\lambda_{y_{11}} = 0.7$	$\lambda_{y_{12}} = 0.7$	$\lambda_{y_{13}} = 0.7$	$\lambda_{y_{21}} = 0.5$	$\lambda_{y_{22}} = 0.7$	$\lambda_{y_{23}} = 0.9$	Load.	CompCor	notc	Boot
Symmetric	PLS	2	0.3549	0.1053	0.3246	0.8005	0.7453	0.6645	0.7420	0.7408	0.7405	0.5784	0.7545	0.8514	0.00	0.00	0.00	0
			0.0395	0.0465	0.0436	0.0332	0.0417	0.0534	0.0348	0.0349	0.0350	0.0649	0.0406	0.0254				
		3	0.3996	0.1013	0.3684	0.8237	0.7720	0.6990	0.7678	0.7680	0.7680	0.6140	0.7829	0.8689	0.00	0.00	0.00	0
			0.0369	0.0451	0.0415	0.0237	0.0311	0.0410	0.0264	0.0264	0.0264	0.0517	0.0299	0.0176				
		5	0.4326	0.0973	0.4007	0.8474	0.7985	0.7259	0.7941	0.7946	0.7940	0.6443	0.8117	0.8898	0.00	0.00	0.00	0
			0.0359	0.0449	0.0409	0.0179	0.0247	0.0343	0.0215	0.0215	0.0215	0.0443	0.0236	0.0126				
		7	0.4415	0.0945	0.4116	0.8550	0.8079	0.7348	0.8025	0.8023	0.8020	0.6532	0.8211	0.8967	0.00	0.00	0.00	0
			0.0355	0.0447	0.0407	0.0164	0.0229	0.0323	0.0202	0.0203	0.0203	0.0424	0.0219	0.0113				
	OrdPLS	2	0.4582	0.0935	0.4251	0.8624	0.8170	0.7428	0.8126	0.8111	0.8111	0.6618	0.8310	0.9037	0.00	0.00	0.00	0
			0.0486	0.0685	0.0618	0.0270	0.0359	0.0487	0.0320	0.0321	0.0322	0.0626	0.0348	0.0195				
		3	0.4553	0.0900	0.4257	0.8635	0.8173	0.7459	0.8116	0.8115	0.8118	0.6619	0.8320	0.9042	0.00	0.00	0.00	0
			0.0412	0.0553	0.0502	0.0209	0.0286	0.0391	0.0255	0.0255	0.0255	0.0509	0.0273	0.0149				
		5	0.4543	0.0920	0.4235	0.8637	0.8170	0.7449	0.8121	0.8123	0.8119	0.6637	0.8319	0.9044	0.00	0.00	0.00	0
			0.0372	0.0484	0.0439	0.0170	0.0239	0.0335	0.0212	0.0212	0.0212	0.0439	0.0227	0.0117				
		7	0.4531	0.0916	0.4240	0.8639	0.8179	0.7451	0.8122	0.8119	0.8117	0.6637	0.8320	0.9045	0.00	0.00	0.00	0
			0.0361	0.0465	0.0422	0.0159	0.0225	0.0319	0.0200	0.0201	0.0201	0.0421	0.0214	0.0109				
	PLSc	2	0.5890	0.0068	0.5809	0.6512	0.5715	0.4844	0.5706	0.5708	0.5695	0.4043	0.5693	0.7417	0.00	0.00	0.00	6887
			0.0667	0.1226	0.1176	0.0668	0.0689	0.0738	0.0550	0.0550	0.0551	0.0788	0.0669	0.0643				
		3	0.6018	-0.0047	0.6045	0.7090	0.6207	0.5324	0.6203	0.6217	0.6198	0.4413	0.6198	0.7976	0.00	0.00	0.00	7525
			0.0549	0.0995	0.0938	0.0566	0.0596	0.0650	0.0466	0.0465	0.0466	0.0697	0.0565	0.0529				
		5	0.6024	0.0002	0.6013	0.7626	0.6669	0.5711	0.6681	0.6693	0.6673	0.4771	0.6663	0.8568	1.00	0.00	0.00	19029
			0.0487	0.0848	0.0792	0.0501	0.0537	0.0601	0.0418	0.0418	0.0418	0.0649	0.0506	0.0448				
		7	0.6012	-0.0008	0.6025	0.7793	0.6831	0.5838	0.6826	0.6826	0.6818	0.4870	0.6808	0.8764	3.00	0.00	0.00	30651
			0.0470	0.0811	0.0755	0.0482	0.0520	0.0586	0.0403	0.0403	0.0403	0.0632	0.0487	0.0421				
	OrdPLSc	2	0.6067	-0.0015	0.6111	0.7935	0.6998	0.5970	0.6992	0.6995	0.6972	0.5032	0.6996	0.8828	99.00	0.00	0.00	199431
			0.0662	0.1339	0.1253	0.0749	0.0810	0.0885	0.0643	0.0644	0.0645	0.0948	0.0776	0.0642				
		3	0.5971	-0.0018	0.6014	0.8006	0.7052	0.6064	0.7032	0.7054	0.7034	0.5079	0.7065	0.8911	48.00	0.00	0.00	83035
			0.0639	0.0997	0.1009	0.0600	0.0689	0.0779	0.0563	0.0558	0.0563	0.0844	0.0679	0.0490				
		5	0.6006	0.0013	0.5998	0.7999	0.7002	0.6007	0.7017	0.7027	0.7010	0.5031	0.7009	0.8948	19.00	0.00	0.00	55107
			0.0561	0.0856	0.0852	0.0518	0.0596	0.0678	0.0478	0.0478	0.0479	0.0741	0.0584	0.0427				
		7	0.5996	0.0001	0.6013	0.7998	0.7012	0.6003	0.7009	0.7013	0.7002	0.5012	0.7002	0.8966	14.00	0.00	0.00	48640
			0.0538	0.0817	0.0811	0.0492	0.0568	0.0650	0.0454	0.0454	0.0454	0.0710	0.0553	0.0406				
	WLSMV	2	0.6061	-0.0017	0.6069	0.7965	0.7024	0.5990	0.7031	0.7002	0.6998	0.5035	0.7042	0.8915	49.00	0.00	0.00	50.00
			0.0652	0.1177	0.1114	0.0578	0.0582	0.0607	0.0531	0.0532	0.0533	0.0622	0.0563	0.0575				
		3	0.6045	-0.0061	0.6074	0.7990	0.7018	0.6019	0.7001	0.7005	0.7001	0.5006	0.7011	0.8974	15.00	0.00	0.00	15.00
			0.0542	0.0944	0.0889	0.0459	0.0469	0.0494	0.0430	0.0429	0.0429	0.0516	0.0455	0.0458				
		5	0.6039	-0.0009	0.6025	0.7985	0.7005	0.5999	0.7006	0.7013	0.7000	0.5014	0.6996	0.8986	0.00	0.00	0.00	0.00
			0.0474	0.0808	0.0753	0.0373	0.0388	0.0420	0.0356	0.0357	0.0357	0.0442	0.0374	0.0363				
		7	0.6025	-0.0014	0.6033	0.7987	0.7019	0.6001	0.7005	0.7003	0.6996	0.5011	0.6995	0.8987	1.00	0.00	0.00	1.00
			0.0453	0.0765	0.0712	0.0346	0.0363	0.0396	0.0336	0.0336	0.0336	0.0420	0.0350	0.0335				
Moderate	PLS	2	0.3463	0.1035	0.3186	0.7964	0.7424	0.6613	0.7380	0.7370	0.7356	0.5733	0.7518	0.8490	0.00	0.00	0.00	0
			0.0409	0.0476	0.0449	0.0358	0.0446	0.0567	0.0374	0.0375	0.0377	0.0686	0.0434	0.0273				
		3	0.4051	0.1013	0.3734	0.8299	0.7786	0.7042	0.7743	0.7755	0.7751	0.6209	0.7906	0.8754	0.00	0.00	0.00	0
			0.0370	0.0453	0.0418	0.0223	0.0298	0.0399	0.0254	0.0252	0.0253	0.0504	0.0286	0.0162				
		5	0.4238	0.0966	0.3950	0.8445	0.7949	0.7211	0.7904	0.7901	0.7894	0.6366	0.8087	0.8880	0.00	0.00	0.00	0
			0.0370	0.0456	0.0420	0.0193	0.0264	0.0365	0.0230	0.0230	0.0231	0.0470	0.0252	0.0135				
		7	0.4352	0.0953	0.4055	0.8516	0.8028	0.7306	0.7985	0.7980	0.7977	0.6472	0.8173	0.8941	0.00	0.00	0.00	0
			0.0364	0.0453	0.0416	0.0175	0.0245	0.0341	0.0214	0.0214	0.0215	0.0444	0.0232	0.0121				
	OrdPLS	2	0.4567	0.0923	0.4257	0.8611	0.8175	0.7439	0.8117	0.8103	0.8094	0.6619	0.8310	0.9032	0.00	0.00	0.00	0
			0.0506	0.0718	0.0645	0.0286	0.0375	0.0509	0.0337	0.0338	0.0340	0.0654	0.0365	0.0206				
		3	0.4547	0.0921	0.4236	0.8630	0.8168	0.7449	0.8115	0.8126	0.8124	0.6634	0.8316	0.9043	0.00	0.00	0.00	0
			0.0408	0.0542	0.0493	0.0201	0.0277	0.0382	0.0247	0.0244	0.0246	0.0496	0.0265	0.0141				
		5	0.4533	0.0913	0.4245	0.8636	0.8174	0.7454	0.8123	0.8121	0.8112	0.6629	0.8322	0.9044	0.00	0.00	0.00	0
			0.0381	0.0497	0.0451	0.0177	0.0247	0.0346	0.0220	0.0220	0.0220	0.0453	0.0234	0.0122				
		7	0.4534	0.0919	0.4236	0.8638	0.8169	0.7455	0.8122	0.8119	0.8117	0.6637	0.8321	0.9045	0.00	0.00	0.00	0
			0.0365	0.0471	0.0428	0.0163	0.0230	0.0324	0.0204	0.0204	0.0205	0.0427	0.0218	0.0111				
	PLSc	2	0.5829	0.0055	0.5785	0.6420	0.5653	0.4803	0.5630	0.5629	0.5605	0.3996	0.5647	0.7322	0.00	0.00	0.00	6114
			0.0700	0.1291	0.1241	0.0703	0.0725	0.0771	0.0578	0.0579	0.0579	0.0818	0.0701	0.0678				
		3	0.5972	0.0026	0.5938	0.7229	0.6328	0.5395	0.6330	0.6349	0.6329	0.4513	0.6309	0.8154	1.00	0.00	0.00	10166
			0.0541	0.0950	0.0896	0.0557	0.0588	0.0646	0.0460	0.0459	0.0460	0.0694	0.0558	0.0514				
		5	0.5965	-0.0007	0.5985	0.7555	0.6600	0.5654	0.6607	0.6616	0.6595	0.4692	0.6600	0.8513	1.00	0.00	0.00	20152
			0.0509	0.0876	0.0823	0.0528	0.0563	0.0626	0.0440	0.0440	0.0439	0.0673	0.0529	0.0471				
		7	0.5992	-0.0002	0.5997	0.7716	0.6743	0.5777	0.6758	0.6755	0.6739	0.4807	0.6739	0.8691	1.00	0.00	0.00	29261
			0.0488	0.0838	0.0784	0.0502	0.0540	0.0604	0.0420	0.0421	0.0420	0.0653	0.0507	0.0442				
	OrdPLSc	2	0.6061	-0.0040	0.6123	0.7891	0.7018	0.5964	0.6981	0.6981	0.6940	0.5024	0.7015	0.8799	88.00	0.00	0.00	202684
			0.0688	0.1421	0.1328	0.0780	0.0844	0.0928	0.0672	0.0674	0.0676	0.0992	0.0814	0.0670				
		3	0.5973	0.0029	0.5971	0.7996	0.7032	0.6021	0.7029	0.7055	0.7034	0.5073	0.7030	0.8926	37.00	0.00	0.00	78073
			0.0628	0.0970	0.0976	0.0586	0.0674	0.0768	0.0547	0.0545	0.0549	0.0830	0.0666	0.0476				
		5	0.5980	0.0003	0.6009	0.7997	0.7016	0.6035	0.7026	0.7037	0.7015	0.5049	0.7028	0.8942	29.00	0.00	0.00	58129
			0.0574	0.0878	0.0876	0.0534	0.0614	0.0695	0.0492	0.0490	0.0492	0.0757	0.0597	0.0435				
		7	0.6001	0.0004	0.6005	0.8000	0.7002	0.6001	0.7015	0.7016	0.7002	0.5017	0.7003	0.8965	15.00	0.00	0.00	51433
			0.0545	0.0829	0.0823	0.0502	0.0576	0.0660	0.0462	0.0462	0.0463	0.0721	0.0564	0.0412				
	WLSMV	2	0.6053	-0.0033	0.6088	0.7947	0.7034	0.6012	0.7007	0.6994	0.6980	0.5024	0.7046	0.8897	49.00	0.00	0.00	49.00
			0.0679	0.1231	0.1165	0.0600	0.0604	0.0629	0.0554	0.0555	0.0555	0.0647	0.0585	0.0598				
		3	0.6037	-0.0007	0.6023	0.7970	0.7003	0.6010	0.7001	0.7021	0.7011	0.5019	0.7005	0.8979	11.00	0.00	0.00	11.00
			0.0533	0.0921	0.0863	0.0440	0.0452	0.0481	0.0415	0.0414	0.0415	0.0502	0.0436	0.0432				
		5	0.6025	-0.0023	0.6044	0.7987	0.7013	0.6006	0.7006	0.7011	0.6989	0.5004	0.7005	0.8980	6.00	0.00	0.00	6.00
			0.0484	0.0826	0.0772	0.0385	0.0399	0.0431	0.0369	0.0368	0.0368	0.0454	0.0385	0.0376				
		7	0.6028	-0.0009	0.6026	0.7988	0.7002	0.6005	0.7008	0.7004	0.6992	0.5011	0.6998	0.8987	2.00	0.00	0.00	2.00
			0.0455	0.0771	0.0718	0.0354	0.0369	0.0400	0.0340	0.0340	0.0341	0.0423	0.0355	0.0343				

Table 2: Results for the model with three common factors

Threshold dist.	Estimator	# cat	γ_1=0.6	$\gamma_2 = 0.0$	$\beta = 0.6$	$\lambda_{x_1} = 0.8$	$\lambda_{x_2} = 0.7$	$\lambda_{x_3} = 0.6$	$\lambda_{y_{11}} = 0.7$	$\lambda_{y_{12}} = 0.7$	$\lambda_{y_{13}} = 0.7$	$\lambda_{y_{21}} = 0.5$	$\lambda_{y_{22}} = 0.7$	$\lambda_{y_{23}} = 0.9$	Load.	CompCor	notc	Boot
Ext. Asymm.	PLS	2	0.2966	0.0902	0.2842	0.7817	0.7124	0.6269	0.7114	0.7113	0.7121	0.5240	0.7228	0.8409	0.00	0.00	0.00	0
			0.0517	0.0568	0.0555	0.0613	0.0754	0.0910	0.0608	0.0610	0.0607	0.1041	0.0722	0.0468				
		3	0.3539	0.0957	0.3321	0.8119	0.7523	0.6701	0.7477	0.7482	0.7486	0.5760	0.7667	0.8639	0.00	0.00	0.00	0
			0.0451	0.0509	0.0493	0.0354	0.0460	0.0593	0.0387	0.0387	0.0386	0.0719	0.0437	0.0257				
		5	0.3991	0.0967	0.3708	0.8345	0.7819	0.7036	0.7767	0.7758	0.7766	0.6139	0.7972	0.8817	0.00	0.00	0.00	0
			0.0401	0.0476	0.0450	0.0237	0.0320	0.0434	0.0277	0.0279	0.0276	0.0552	0.0306	0.0167				
		7	0.4159	0.0959	0.3872	0.8443	0.7932	0.7166	0.7881	0.7875	0.7879	0.6296	0.8088	0.8894	0.00	0.00	0.00	0
			0.0387	0.0468	0.0437	0.0204	0.0282	0.0390	0.0246	0.0247	0.0246	0.0502	0.0269	0.0142				
	OrdPLS	2	0.4586	0.0801	0.4416	0.8620	0.8126	0.7421	0.8080	0.8078	0.8092	0.6529	0.8291	0.9028	0.00	0.00	0.00	0
			0.0676	0.1087	0.0910	0.0439	0.0582	0.0768	0.0486	0.0487	0.0483	0.0994	0.0571	0.0344				
		3	0.4564	0.0888	0.4293	0.8623	0.8157	0.7443	0.8099	0.8102	0.8109	0.6603	0.8312	0.9035	0.00	0.00	0.00	0
			0.0514	0.0720	0.0642	0.0275	0.0373	0.0510	0.0331	0.0331	0.0329	0.0660	0.0356	0.0194				
		5	0.4556	0.0914	0.4247	0.8630	0.8169	0.7447	0.8118	0.8108	0.8112	0.6615	0.8321	0.9040	0.00	0.00	0.00	0
			0.0423	0.0561	0.0509	0.0203	0.0280	0.0391	0.0250	0.0251	0.0250	0.0515	0.0268	0.0140				
		7	0.4540	0.0923	0.4235	0.8633	0.8169	0.7447	0.8117	0.8112	0.8114	0.6625	0.8319	0.9041	0.00	0.00	0.00	0
			0.0393	0.0513	0.0466	0.0180	0.0253	0.0356	0.0225	0.0226	0.0225	0.0468	0.0241	0.0124				
	PLSc	2	0.5514	-0.0144	0.5726	0.5990	0.5135	0.4325	0.5145	0.5140	0.5130	0.3485	0.5124	0.6939	3.00	0.00	0.00	42061
			0.0980	0.1930	0.1873	0.1004	0.1021	0.1059	0.0811	0.0813	0.0815	0.1074	0.0990	0.0961				
		3	0.5669	0.0005	0.5694	0.6763	0.5846	0.4946	0.5838	0.5851	0.5836	0.4072	0.5858	0.7733	0.00	0.00	0.00	18581
			0.0723	0.1232	0.1196	0.0754	0.0782	0.0840	0.0622	0.0622	0.0622	0.0880	0.0749	0.0702				
		5	0.5835	0.0025	0.5803	0.7302	0.6374	0.5427	0.6368	0.6357	0.6363	0.4482	0.6377	0.8299	1.00	0.00	0.00	21914
			0.0580	0.0971	0.0926	0.0602	0.0635	0.0698	0.0501	0.0502	0.0501	0.0751	0.0605	0.0546				
		7	0.5881	0.0026	0.5859	0.7535	0.6568	0.5602	0.6571	0.6567	0.6566	0.4650	0.6571	0.8531	2.00	0.00	0.00	27152
			0.0537	0.0900	0.0852	0.0555	0.0593	0.0658	0.0465	0.0466	0.0465	0.0707	0.0561	0.0494				
	OrdPLSc	2	0.6072	-0.0388	0.6550	0.7894	0.6937	0.5934	0.6971	0.6951	0.6909	0.4927	0.6971	0.8638	212.00	1.00	0.00	465423
			0.0930	0.2345	0.2140	0.1062	0.1195	0.1332	0.0933	0.0932	0.0939	0.1446	0.1166	0.0938				
		3	0.5926	-0.0027	0.6038	0.8009	0.7069	0.6055	0.7060	0.7054	0.7048	0.5125	0.7082	0.8845	76.00	0.00	0.00	119120
			0.0783	0.1322	0.1319	0.0721	0.0844	0.0973	0.0693	0.0696	0.0694	0.1049	0.0831	0.0586				
		5	0.6000	-0.0019	0.6025	0.7980	0.7029	0.6021	0.7019	0.7013	0.7022	0.5037	0.7023	0.8924	33.00	0.00	0.00	77407
			0.0643	0.1010	0.1008	0.0593	0.0685	0.0779	0.0555	0.0556	0.0554	0.0852	0.0673	0.0479				
		7	0.5994	0.0009	0.6000	0.7993	0.7014	0.6018	0.7022	0.7021	0.7012	0.5044	0.7020	0.8937	28.00	0.00	0.00	59205
			0.0589	0.0909	0.0905	0.0543	0.0625	0.0713	0.0501	0.0502	0.0504	0.0777	0.0609	0.0442				
	WLSMV	2	0.6054	-0.0265	0.6404	0.7972	0.7003	0.6023	0.6991	0.6979	0.7006	0.5022	0.7073	0.8822	115.00	1.00	0.00	130.00
			0.0902	0.1781	0.1681	0.0799	0.0800	0.0828	0.0730	0.0730	0.0729	0.0850	0.0770	0.0791				
		3	0.6055	-0.0108	0.6150	0.7971	0.7003	0.6013	0.6983	0.6985	0.7001	0.5019	0.7038	0.8927	33.00	0.00	0.00	34.00
			0.0677	0.1217	0.1144	0.0573	0.0583	0.0615	0.0536	0.0537	0.0535	0.0638	0.0559	0.0560				
		5	0.6057	-0.0042	0.6064	0.7973	0.7007	0.6005	0.7001	0.6992	0.6992	0.5002	0.7012	0.8963	12.00	0.00	0.00	12.00
			0.0545	0.0947	0.0885	0.0437	0.0452	0.0487	0.0418	0.0418	0.0417	0.0511	0.0435	0.0425				
		7	0.6040	-0.0015	0.6036	0.7980	0.7003	0.6000	0.6999	0.6998	0.6993	0.5008	0.7001	0.8973	6.00	0.00	0.00	6.00
			0.0500	0.0856	0.0799	0.0391	0.0408	0.0442	0.0377	0.0377	0.0377	0.0466	0.0392	0.0379				
Alt. Mod. Asym.	PLS	2	0.3443	0.1062	0.3166	0.7904	0.7279	0.6600	0.7325	0.7277	0.7311	0.5788	0.7320	0.8387	0.00	0.00	0.00	0
			0.0392	0.0467	0.0430	0.0360	0.0457	0.0561	0.0366	0.0373	0.0368	0.0671	0.0445	0.0285				
		3	0.4048	0.1013	0.3733	0.8299	0.7780	0.7045	0.7747	0.7745	0.7742	0.6208	0.7897	0.8746	0.00	0.00	0.00	0
			0.0370	0.0453	0.0417	0.0223	0.0297	0.0398	0.0253	0.0253	0.0254	0.0504	0.0286	0.0163				
		5	0.4235	0.0992	0.3923	0.8403	0.7863	0.7199	0.7872	0.7827	0.7872	0.6388	0.7977	0.8820	0.00	0.00	0.00	0
			0.0359	0.0450	0.0408	0.0192	0.0263	0.0357	0.0225	0.0225	0.0225	0.0459	0.0251	0.0137				
		7	0.4353	0.0967	0.4041	0.8495	0.7980	0.7296	0.7970	0.7936	0.7962	0.6484	0.8107	0.8907	0.00	0.00	0.00	0
			0.0356	0.0448	0.0407	0.0173	0.0241	0.0336	0.0209	0.0210	0.0210	0.0437	0.0231	0.0121				
	OrdPLS	2	0.4564	0.0907	0.4276	0.8624	0.8175	0.7433	0.8119	0.8115	0.8105	0.6636	0.8294	0.9035	0.00	0.00	0.00	0
			0.0500	0.0712	0.0640	0.0288	0.0384	0.0509	0.0338	0.0345	0.0341	0.0646	0.0375	0.0213				
		3	0.4545	0.0920	0.4236	0.8633	0.8170	0.7453	0.8121	0.8122	0.8117	0.6631	0.8314	0.9040	0.00	0.00	0.00	0
			0.0407	0.0542	0.0493	0.0201	0.0277	0.0381	0.0245	0.0246	0.0247	0.0496	0.0265	0.0142				
		5	0.4538	0.0912	0.4241	0.8638	0.8179	0.7447	0.8121	0.8122	0.8122	0.6632	0.8320	0.9045	0.00	0.00	0.00	0
			0.0379	0.0496	0.0450	0.0178	0.0247	0.0347	0.0220	0.0221	0.0220	0.0452	0.0236	0.0123				
		7	0.4536	0.0919	0.4234	0.8641	0.8173	0.7450	0.8124	0.8118	0.8117	0.6638	0.8318	0.9045	0.00	0.00	0.00	0
			0.0364	0.0471	0.0427	0.0162	0.0230	0.0324	0.0204	0.0204	0.0205	0.0427	0.0218	0.0111				
	PLSc	2	0.5977	-0.0031	0.6041	0.6253	0.5512	0.4670	0.5465	0.5566	0.5454	0.3904	0.5445	0.7045	0.00	0.00	0.00	4461
			0.0696	0.1380	0.1316	0.0679	0.0699	0.0750	0.0554	0.0555	0.0555	0.0785	0.0673	0.0658				
		3	0.5973	0.0022	0.5951	0.7231	0.6317	0.5401	0.6323	0.6340	0.6316	0.4505	0.6307	0.8127	0.00	0.00	0.00	9615
			0.0540	0.0951	0.0897	0.0554	0.0588	0.0645	0.0458	0.0459	0.0460	0.0693	0.0558	0.0515				
		5	0.6045	-0.0029	0.6074	0.7434	0.6541	0.5575	0.6503	0.6582	0.6502	0.4641	0.6510	0.8345	0.00	0.00	0.00	11732
			0.0502	0.0895	0.0836	0.0514	0.0550	0.0610	0.0424	0.0428	0.0425	0.0655	0.0517	0.0467				
		7	0.6038	-0.0019	0.6052	0.7653	0.6712	0.5729	0.6702	0.6733	0.6684	0.4778	0.6686	0.8591	1.00	0.00	0.00	21020
			0.0481	0.0843	0.0785	0.0493	0.0530	0.0594	0.0409	0.0412	0.0409	0.0641	0.0498	0.0439				
	OrdPLSc	2	0.6073	-0.0095	0.6198	0.7926	0.6967	0.5980	0.6975	0.6994	0.6976	0.5057	0.6993	0.8800	119.00	0.00	0.00	233367
			0.0685	0.1433	0.1344	0.0786	0.0862	0.0923	0.0677	0.0681	0.0679	0.0978	0.0826	0.0679				
		3	0.5964	0.0031	0.5973	0.8011	0.7027	0.6030	0.7035	0.7045	0.7034	0.5073	0.7034	0.8907	39.00	0.00	0.00	75784
			0.0625	0.0966	0.0971	0.0584	0.0676	0.0764	0.0545	0.0545	0.0547	0.0828	0.0663	0.0480				
		5	0.5986	-0.0002	0.6005	0.7995	0.7028	0.6019	0.7017	0.7039	0.7013	0.5035	0.7015	0.8948	24.00	0.00	0.00	61201
			0.0577	0.0881	0.0882	0.0537	0.0616	0.0701	0.0497	0.0495	0.0498	0.0764	0.0604	0.0439				
		7	0.5999	0.0011	0.5996	0.8003	0.7018	0.5996	0.7021	0.7016	0.7006	0.5022	0.7011	0.8958	18.00	0.00	0.00	49995
			0.0544	0.0828	0.0821	0.0501	0.0573	0.0659	0.0461	0.0460	0.0462	0.0719	0.0561	0.0412				
	WLSMV	2	0.6039	-0.0076	0.6117	0.7965	0.7036	0.6009	0.7025	0.7025	0.6992	0.5052	0.7028	0.8922	52.00	0.00	0.00	56.00
			0.0673	0.1224	0.1163	0.0616	0.0621	0.0630	0.0557	0.0567	0.0559	0.0642	0.0603	0.0626				
		3	0.6035	-0.0007	0.6023	0.7981	0.7009	0.6013	0.7011	0.7014	0.6998	0.5016	0.7000	0.8975	5.00	0.00	0.00	5.00
			0.0532	0.0919	0.0863	0.0440	0.0452	0.0481	0.0414	0.0415	0.0416	0.0502	0.0437	0.0434				
		5	0.6023	-0.0020	0.6028	0.7990	0.7020	0.5996	0.7004	0.7014	0.7006	0.5007	0.7002	0.8985	7.00	0.00	0.00	7.00
			0.0483	0.0825	0.0771	0.0388	0.0402	0.0431	0.0369	0.0370	0.0369	0.0453	0.0388	0.0380				
		7	0.6032	-0.0011	0.6026	0.7993	0.7008	0.5998	0.7010	0.7002	0.6994	0.5014	0.6996	0.8984	3.00	0.00	0.00	3.00
			0.0455	0.0771	0.0719	0.0353	0.0369	0.0400	0.0340	0.0341	0.0340	0.0423	0.0356	0.0343				

Table 3: Results for the model with three common factors

Threshold dist.	Estimator	# cat	γ_1=0.6	$\gamma_2 = 0.0$	$\beta = 0.6$	$\lambda_{x_1} = 0.8$	$\lambda_{x_2} = 0.7$	$\lambda_{x_3} = 0.6$	$\lambda_{y_{11}} = 0.7$	$\lambda_{y_{12}} = 0.7$	$\lambda_{y_{13}} = 0.7$	$\lambda_{y_{21}} = 0.5$	$\lambda_{y_{22}} = 0.7$	$\lambda_{y_{23}} = 0.9$	Load.	CompCor	notc	Boot
Alt. Ext. Asym.	PLS	2	0.2783	0.1093	0.2586	0.7366	0.6447	0.6230	0.6731	0.6724	0.6722	0.5431	0.6450	0.7748	0.00	0.00	0.00	0
			0.0413	0.0505	0.0443	0.0670	0.0862	0.0849	0.0596	0.0700	0.0599	0.0971	0.0861	0.0617				
		3	0.3461	0.1122	0.3182	0.7806	0.6946	0.6665	0.7246	0.7072	0.7245	0.5919	0.6969	0.8179	0.00	0.00	0.00	0
			0.0379	0.0469	0.0415	0.0378	0.0507	0.0555	0.0367	0.0407	0.0368	0.0663	0.0497	0.0324				
		5	0.3963	0.1057	0.3644	0.8190	0.7505	0.7016	0.7662	0.7513	0.7654	0.6220	0.7581	0.8592	0.00	0.00	0.00	0
			0.0367	0.0458	0.0412	0.0242	0.0332	0.0417	0.0264	0.0274	0.0266	0.0526	0.0321	0.0188				
		7	0.4145	0.1025	0.3825	0.8332	0.7711	0.7149	0.7808	0.7690	0.7802	0.6351	0.7808	0.8736	0.00	0.00	0.00	0
			0.0362	0.0454	0.0410	0.0206	0.0285	0.0377	0.0236	0.0241	0.0237	0.0482	0.0275	0.0152				
	OrdPLS	2	0.4573	0.0692	0.4508	0.8824	0.8509	0.7379	0.8175	0.8279	0.8176	0.6423	0.8699	0.9258	0.00	0.00	0.00	0
			0.0808	0.1222	0.1201	0.0502	0.0656	0.0808	0.0582	0.0667	0.0584	0.0987	0.0627	0.0400				
		3	0.4578	0.0885	0.4323	0.8641	0.8171	0.7434	0.8125	0.8121	0.8123	0.6628	0.8309	0.9041	0.00	0.00	0.00	0
			0.0489	0.0697	0.0627	0.0290	0.0396	0.0502	0.0336	0.0358	0.0337	0.0633	0.0387	0.0224				
		5	0.4552	0.0914	0.4249	0.8637	0.8179	0.7442	0.8126	0.8116	0.8121	0.6621	0.8312	0.9043	0.00	0.00	0.00	0
			0.0411	0.0550	0.0498	0.0207	0.0287	0.0389	0.0250	0.0258	0.0252	0.0505	0.0276	0.0148				
		7	0.4542	0.0921	0.4235	0.8635	0.8181	0.7447	0.8120	0.8116	0.8120	0.6630	0.8317	0.9042	0.00	0.00	0.00	0
			0.0387	0.0507	0.0460	0.0183	0.0255	0.0355	0.0225	0.0229	0.0226	0.0464	0.0245	0.0128				
	PLSc	2	0.6663	-0.0886	0.7702	0.4806	0.4366	0.3620	0.4106	0.4654	0.4099	0.2905	0.4222	0.5171	0.00	31.00	0.00	157661
			0.0992	0.3259	0.3071	0.0845	0.0789	0.0869	0.0662	0.0659	0.0663	0.0867	0.0755	0.0839				
		3	0.6380	-0.0329	0.6783	0.5912	0.5303	0.4423	0.5111	0.5527	0.5115	0.3642	0.5200	0.6461	0.00	0.00	0.00	5627
			0.0709	0.1706	0.1611	0.0660	0.0658	0.0727	0.0528	0.0533	0.0528	0.0751	0.0627	0.0642				
		5	0.6125	-0.0072	0.6219	0.6891	0.6110	0.5156	0.6010	0.6216	0.5998	0.4272	0.6042	0.7674	0.00	0.00	0.00	4047
			0.0564	0.1079	0.1012	0.0564	0.0591	0.0652	0.0459	0.0467	0.0460	0.0695	0.0561	0.0533				
		7	0.6080	-0.0034	0.6126	0.7241	0.6399	0.5409	0.6321	0.6468	0.6307	0.4503	0.6344	0.8092	0.00	0.00	0.00	7477
			0.0525	0.0958	0.0897	0.0530	0.0563	0.0626	0.0434	0.0442	0.0435	0.0670	0.0533	0.0491				
	OrdPLSc	2	0.5858	-0.0287	0.6369	0.7962	0.7153	0.6311	0.7271	0.7087	0.7229	0.5413	0.7212	0.8535	967.00	15.00	0.00	4484252
			0.1122	0.3018	0.2912	0.1188	0.1384	0.1421	0.1134	0.1160	0.1121	0.1570	0.1403	0.1094				
		3	0.5863	0.0030	0.5976	0.8060	0.7116	0.6152	0.7136	0.7119	0.7143	0.5260	0.7135	0.8819	117.00	0.00	0.00	146370
			0.0771	0.1286	0.1315	0.0712	0.0867	0.0937	0.0698	0.0718	0.0695	0.1009	0.0857	0.0602				
		5	0.5957	0.0026	0.5976	0.8016	0.7059	0.6047	0.7070	0.7047	0.7054	0.5090	0.7044	0.8921	53.00	0.00	0.00	84468
			0.0637	0.0984	0.0995	0.0592	0.0696	0.0775	0.0556	0.0564	0.0558	0.0842	0.0684	0.0487				
		7	0.5981	0.0024	0.5986	0.8003	0.7032	0.6016	0.7038	0.7023	0.7027	0.5057	0.7016	0.8940	31.00	0.00	0.00	64161
			0.0590	0.0900	0.0902	0.0547	0.0634	0.0719	0.0507	0.0511	0.0507	0.0778	0.0621	0.0449				
	WLSMV	2	0.6217	-0.0424	0.6785	0.7846	0.6861	0.6087	0.7016	0.6860	0.6941	0.5148	0.6850	0.8495	175.00	7.00	0.00	207.00
			0.1016	0.2187	0.2208	0.1030	0.1123	0.0911	0.0831	0.0953	0.0831	0.0902	0.1171	0.1159				
		3	0.6032	-0.0051	0.6142	0.8001	0.7029	0.6013	0.7035	0.7034	0.7023	0.5051	0.7030	0.8903	80.00	0.00	0.00	84.00
			0.0658	0.1182	0.1131	0.0628	0.0646	0.0618	0.0551	0.0583	0.0553	0.0626	0.0634	0.0659				
		5	0.6041	-0.0035	0.6053	0.7987	0.7028	0.5999	0.7020	0.7008	0.7004	0.5007	0.7003	0.8969	22.00	0.00	0.00	22.00
			0.0533	0.0928	0.0872	0.0454	0.0469	0.0484	0.0420	0.0430	0.0421	0.0505	0.0456	0.0460				
		7	0.6037	-0.0010	0.6031	0.7981	0.7025	0.6004	0.7008	0.7005	0.7004	0.5013	0.6999	0.8977	9.00	0.00	0.00	9.00
			0.0493	0.0846	0.0792	0.0399	0.0415	0.0440	0.0378	0.0383	0.0378	0.0462	0.0402	0.0396				

Table 4: Results for the model with two composites and one common factor

Threshold dist.	Estimator	# cat	γ_1=0.6	$\gamma_2 = 0.0$	$\beta = 0.6$	$\lambda_{y_4} = 0.5$	$\lambda_{y_5} = 0.7$	$\lambda_{y_6} = 0.9$	$w_{x_1} = 0.3$	$w_{x_2} = 0.5$	$w_{x_3} = 0.6$	$w_{y_{11}} = 0.4$	$w_{y_{12}} = 0.5$	$w_{y_{13}} = 0.5$	Load.	CompCor	notc	Boot
Symmetric	PLS	2	0.4451	0.0757	0.3767	0.5796	0.7553	0.8515	0.2995	0.5556	0.6051	0.4552	0.5094	0.5204	0.00	0.00	0.00	0
			0.0364	0.0484	0.0443	0.0605	0.0376	0.0227	0.0927	0.0869	0.0832	0.0707	0.0666	0.0681				
		3	0.5107	0.0601	0.4368	0.6160	0.7830	0.8686	0.3014	0.5310	0.6026	0.4353	0.5040	0.5095	0.00	0.00	0.00	0
			0.0327	0.0474	0.0425	0.0483	0.0277	0.0158	0.0785	0.0756	0.0712	0.0592	0.0553	0.0568				
		5	0.5670	0.0290	0.4953	0.6443	0.8120	0.8897	0.3005	0.5113	0.5994	0.4134	0.5019	0.5029	0.00	0.00	0.00	0
			0.0301	0.0478	0.0425	0.0417	0.0219	0.0112	0.0701	0.0700	0.0654	0.0530	0.0485	0.0506				
		7	0.5830	0.0171	0.5146	0.6542	0.8212	0.8962	0.2985	0.5062	0.5987	0.4078	0.4995	0.5013	0.00	0.00	0.00	0
			0.0294	0.0480	0.0424	0.0399	0.0203	0.0101	0.0680	0.0686	0.0641	0.0513	0.0468	0.0490				
	OrdPLS	2	0.6104	-0.0059	0.5485	0.6636	0.8314	0.9035	0.2980	0.4963	0.5936	0.3924	0.5019	0.4992	0.00	0.00	0.00	0
			0.0465	0.0907	0.0804	0.0600	0.0332	0.0185	0.1056	0.1173	0.1055	0.0930	0.0761	0.0853				
		3	0.6055	0.0029	0.5413	0.6639	0.8318	0.9042	0.2994	0.4942	0.5980	0.3971	0.5001	0.4984	0.00	0.00	0.00	0
			0.0370	0.0672	0.0597	0.0483	0.0260	0.0138	0.0844	0.0916	0.0829	0.0706	0.0598	0.0656				
		5	0.6048	0.0017	0.5405	0.6638	0.8321	0.9042	0.3001	0.4957	0.5983	0.3973	0.5005	0.4990	0.00	0.00	0.00	0
			0.0313	0.0548	0.0485	0.0417	0.0213	0.0107	0.0720	0.0756	0.0695	0.0568	0.0500	0.0536				
		7	0.6032	0.0015	0.5396	0.6648	0.8320	0.9041	0.2981	0.4979	0.5980	0.3990	0.4988	0.4993	0.00	0.00	0.00	0
			0.0299	0.0515	0.0455	0.0397	0.0200	0.0098	0.0688	0.0714	0.0661	0.0532	0.0475	0.0504				
	PLSc	2	0.4451	0.0942	0.4685	0.4056	0.5712	0.7406	0.2995	0.5556	0.6051	0.4552	0.5094	0.5204	0.00	0.00	0.00	2137
			0.0364	0.0602	0.0567	0.0712	0.0607	0.0580	0.0927	0.0870	0.0832	0.0707	0.0666	0.0680				
		3	0.5107	0.0717	0.5199	0.4452	0.6207	0.7951	0.3013	0.5311	0.6025	0.4353	0.5040	0.5096	1.00	0.00	0.00	2483
			0.0327	0.0565	0.0514	0.0622	0.0507	0.0468	0.0786	0.0757	0.0712	0.0593	0.0553	0.0568				
		5	0.5670	0.0332	0.5666	0.4784	0.6665	0.8558	0.3005	0.5113	0.5994	0.4134	0.5019	0.5029	0.00	0.00	0.00	7010
			0.0300	0.0547	0.0493	0.0573	0.0445	0.0391	0.0701	0.0699	0.0654	0.0529	0.0485	0.0506				
		7	0.5830	0.0193	0.5822	0.4900	0.6816	0.8732	0.2985	0.5062	0.5987	0.4078	0.4995	0.5013	0.00	0.00	0.00	12623
			0.0293	0.0544	0.0487	0.0558	0.0428	0.0368	0.0680	0.0685	0.0640	0.0512	0.0469	0.0490				
	OrdPLSc	2	0.6101	-0.0065	0.6138	0.5030	0.6999	0.8846	0.2976	0.4963	0.5943	0.3917	0.5024	0.4996	62.00	0.00	0.00	118064
			0.0467	0.1023	0.0920	0.0877	0.0718	0.0601	0.1058	0.1176	0.1057	0.0933	0.0764	0.0856				
		3	0.6054	0.0038	0.6036	0.5077	0.7035	0.8906	0.2998	0.4940	0.5982	0.3970	0.4998	0.4988	29.00	0.00	0.00	54083
			0.0370	0.0748	0.0684	0.0758	0.0610	0.0456	0.0842	0.0917	0.0829	0.0705	0.0598	0.0655				
		5	0.6049	0.0020	0.6035	0.5025	0.6985	0.8947	0.3003	0.4953	0.5986	0.3974	0.5006	0.4989	5.00	0.00	0.00	31682
			0.0313	0.0611	0.0555	0.0645	0.0506	0.0385	0.0719	0.0757	0.0695	0.0567	0.0499	0.0535				
		7	0.6033	0.0016	0.6028	0.5040	0.6995	0.8937	0.2983	0.4978	0.5979	0.3993	0.4988	0.4991	7.00	0.00	0.00	21461
			0.0299	0.0575	0.0520	0.0607	0.0468	0.0364	0.0689	0.0715	0.0661	0.0532	0.0474	0.0504				
Moderate	PLS	2	0.4338	0.0768	0.3695	0.5715	0.7520	0.8497	0.2962	0.5560	0.6093	0.4611	0.5077	0.5208	0.00	0.00	0.00	0
			0.0379	0.0493	0.0455	0.0644	0.0400	0.0244	0.0971	0.0915	0.0873	0.0741	0.0705	0.0715				
		3	0.5242	0.0521	0.4488	0.6215	0.7914	0.8752	0.2990	0.5271	0.6024	0.4297	0.5046	0.5077	0.00	0.00	0.00	0
			0.0322	0.0479	0.0429	0.0472	0.0263	0.0144	0.0766	0.0748	0.0703	0.0583	0.0540	0.0558				
		5	0.5554	0.0334	0.4842	0.6377	0.8088	0.8875	0.2988	0.5156	0.6009	0.4176	0.5015	0.5051	0.00	0.00	0.00	0
			0.0315	0.0486	0.0435	0.0442	0.0234	0.0121	0.0729	0.0727	0.0682	0.0556	0.0512	0.0532				
		7	0.5737	0.0225	0.5045	0.6479	0.8178	0.8937	0.2979	0.5090	0.6000	0.4107	0.5003	0.5029	0.00	0.00	0.00	0
			0.0304	0.0486	0.0433	0.0417	0.0216	0.0109	0.0701	0.0707	0.0659	0.0534	0.0488	0.0510				
	OrdPLS	2	0.6084	-0.0044	0.5487	0.6610	0.8311	0.9032	0.2964	0.4916	0.5982	0.3951	0.4996	0.4981	0.00	0.00	0.00	0
			0.0488	0.0956	0.0844	0.0633	0.0349	0.0194	0.1119	0.1242	0.1109	0.0979	0.0806	0.0898				
		3	0.6069	0.0014	0.5411	0.6642	0.8320	0.9041	0.2986	0.4942	0.5988	0.3960	0.5014	0.4981	0.00	0.00	0.00	0
			0.0358	0.0647	0.0576	0.0471	0.0249	0.0130	0.0817	0.0881	0.0799	0.0675	0.0577	0.0629				
		5	0.6043	0.0020	0.5404	0.6634	0.8324	0.9041	0.3002	0.4954	0.5989	0.3973	0.4999	0.4992	0.00	0.00	0.00	0
			0.0324	0.0568	0.0504	0.0431	0.0220	0.0111	0.0743	0.0783	0.0718	0.0590	0.0519	0.0556				
		7	0.6039	0.0019	0.5399	0.6642	0.8326	0.9042	0.2989	0.4964	0.5986	0.3984	0.4993	0.4992	0.00	0.00	0.00	0
			0.0304	0.0526	0.0465	0.0404	0.0204	0.0101	0.0698	0.0729	0.0672	0.0544	0.0483	0.0515				
	PLSc	2	0.4338	0.0963	0.4624	0.3968	0.5650	0.7329	0.2962	0.5560	0.6093	0.4611	0.5077	0.5208	0.00	0.00	0.00	2112
			0.0379	0.0619	0.0585	0.0743	0.0637	0.0611	0.0970	0.0913	0.0872	0.0740	0.0705	0.0715				
		3	0.5242	0.0613	0.5277	0.4535	0.6326	0.8135	0.2990	0.5271	0.6024	0.4297	0.5046	0.5077	0.00	0.00	0.00	3544
			0.0322	0.0563	0.0514	0.0616	0.0498	0.0453	0.0767	0.0748	0.0703	0.0583	0.0540	0.0558				
		5	0.5554	0.0384	0.5563	0.4718	0.6604	0.8491	0.2988	0.5156	0.6009	0.4176	0.5015	0.5051	0.00	0.00	0.00	7114
			0.0315	0.0559	0.0508	0.0597	0.0469	0.0413	0.0730	0.0728	0.0683	0.0555	0.0512	0.0531				
		7	0.5737	0.0256	0.5733	0.4835	0.6757	0.8655	0.2980	0.5088	0.6001	0.4108	0.5004	0.5029	1.00	0.00	0.00	10344
			0.0305	0.0553	0.0499	0.0576	0.0447	0.0387	0.0701	0.0707	0.0660	0.0534	0.0488	0.0510				
	OrdPLSc	2	0.6096	-0.0020	0.6127	0.4983	0.7018	0.8820	0.2977	0.4922	0.5969	0.3956	0.4994	0.4975	68.00	0.00	0.00	125118
			0.0489	0.1082	0.0968	0.0922	0.0751	0.0626	0.1119	0.1243	0.1112	0.0982	0.0807	0.0900				
		3	0.6070	0.0017	0.6038	0.5080	0.7013	0.8918	0.2989	0.4948	0.5982	0.3957	0.5013	0.4985	21.00	0.00	0.00	46862
			0.0359	0.0720	0.0658	0.0734	0.0590	0.0441	0.0816	0.0882	0.0801	0.0676	0.0577	0.0630				
		5	0.6046	0.0021	0.6034	0.5033	0.7005	0.8937	0.3001	0.4955	0.5988	0.3975	0.4998	0.4991	12.00	0.00	0.00	33044
			0.0324	0.0634	0.0576	0.0665	0.0521	0.0396	0.0743	0.0783	0.0719	0.0589	0.0518	0.0555				
		7	0.6039	0.0022	0.6031	0.5035	0.7001	0.8939	0.2992	0.4962	0.5987	0.3984	0.4989	0.4995	6.00	0.00	0.00	24475
			0.0303	0.0587	0.0531	0.0618	0.0479	0.0371	0.0698	0.0728	0.0671	0.0544	0.0483	0.0514				

Table 5: Results for the model with two composites and one common factor

Threshold dist.	Estimator	# cat	γ_1=0.6	$\gamma_2 = 0.0$	$\beta = 0.6$	$\lambda_{y_4} = 0.5$	$\lambda_{y_5} = 0.7$	$\lambda_{y_6} = 0.9$	$w_{x_1} = 0.3$	$w_{x_2} = 0.5$	$w_{x_3} = 0.6$	$w_{y_{11}} = 0.4$	$w_{y_{12}} = 0.5$	$w_{y_{13}} = 0.5$	Load.	CompCor	notc	Boot
Ext. Asymm.	PLS	2	0.3681	0.0779	0.3193	0.5310	0.7267	0.8392	0.2765	0.5774	0.6123	0.4792	0.5072	0.5320	0.00	0.00	0.00	0
			0.0500	0.0585	0.0561	0.0971	0.0658	0.0420	0.1331	0.1281	0.1244	0.1069	0.1033	0.1036				
		3	0.4523	0.0659	0.3869	0.5779	0.7673	0.8642	0.2870	0.5545	0.6052	0.4520	0.5064	0.5213	0.00	0.00	0.00	0
			0.0416	0.0530	0.0501	0.0676	0.0405	0.0229	0.0996	0.0970	0.0932	0.0787	0.0745	0.0759				
		5	0.5194	0.0457	0.4480	0.6155	0.7969	0.8820	0.2912	0.5304	0.6026	0.4303	0.5038	0.5099	0.00	0.00	0.00	0
			0.0352	0.0503	0.0460	0.0518	0.0285	0.0149	0.0814	0.0807	0.0764	0.0631	0.0587	0.0605				
		7	0.5461	0.0339	0.4752	0.6313	0.8089	0.8892	0.2939	0.5215	0.6000	0.4211	0.5024	0.5061	0.00	0.00	0.00	0
			0.0331	0.0498	0.0450	0.0470	0.0250	0.0126	0.0759	0.0762	0.0719	0.0587	0.0540	0.0562				
	OrdPLS	2	0.6153	-0.0155	0.5629	0.6596	0.8306	0.9023	0.2897	0.4894	0.5950	0.3884	0.4992	0.5014	0.00	0.00	0.00	0
			0.0708	0.1784	0.1515	0.1005	0.0602	0.0398	0.1813	0.1942	0.1761	0.1565	0.1247	0.1410				
		3	0.6077	-0.0021	0.5459	0.6622	0.8314	0.9040	0.2960	0.4948	0.5956	0.3932	0.4998	0.5012	0.00	0.00	0.00	0
			0.0476	0.0919	0.0805	0.0631	0.0338	0.0180	0.1117	0.1205	0.1088	0.0941	0.0790	0.0866				
		5	0.6050	-0.0004	0.5418	0.6631	0.8320	0.9041	0.2969	0.4950	0.5990	0.3958	0.5007	0.4996	0.00	0.00	0.00	0
			0.0367	0.0660	0.0584	0.0487	0.0252	0.0129	0.0846	0.0896	0.0818	0.0683	0.0593	0.0640				
		7	0.6042	0.0010	0.5403	0.6644	0.8321	0.9038	0.2991	0.4958	0.5983	0.3978	0.5002	0.4989	0.00	0.00	0.00	0
			0.0333	0.0585	0.0518	0.0442	0.0225	0.0113	0.0765	0.0804	0.0738	0.0606	0.0532	0.0571				
	PLSc	2	0.3680	0.1017	0.4165	0.3569	0.5198	0.6896	0.2766	0.5772	0.6125	0.4789	0.5071	0.5324	2.00	0.00	0.00	11492
			0.0500	0.0769	0.0753	0.0999	0.0911	0.0886	0.1331	0.1280	0.1242	0.1069	0.1031	0.1035				
		3	0.4523	0.0801	0.4701	0.4101	0.5877	0.7730	0.2870	0.5545	0.6052	0.4520	0.5064	0.5213	0.00	0.00	0.00	8396
			0.0415	0.0646	0.0618	0.0799	0.0680	0.0630	0.0997	0.0971	0.0933	0.0786	0.0744	0.0758				
		5	0.5194	0.0533	0.5221	0.4510	0.6374	0.8299	0.2912	0.5304	0.6026	0.4303	0.5038	0.5099	0.00	0.00	0.00	10865
			0.0351	0.0588	0.0546	0.0669	0.0539	0.0481	0.0815	0.0808	0.0766	0.0631	0.0587	0.0605				
		7	0.5461	0.0390	0.5454	0.4674	0.6578	0.8523	0.2939	0.5215	0.6000	0.4211	0.5024	0.5061	0.00	0.00	0.00	12985
			0.0331	0.0572	0.0525	0.0628	0.0497	0.0433	0.0760	0.0762	0.0719	0.0586	0.0540	0.0562				
	OrdPLSc	2	0.6138	-0.0187	0.6352	0.5050	0.7024	0.8608	0.2942	0.4871	0.5953	0.3897	0.4973	0.5004	134.00	0.00	0.00	192388
			0.0711	0.1970	0.1696	0.1416	0.1136	0.0950	0.1813	0.1949	0.1765	0.1568	0.1251	0.1413				
		3	0.6073	-0.0051	0.6108	0.5119	0.7060	0.8860	0.2966	0.4939	0.5959	0.3944	0.4997	0.5000	50.00	0.00	0.00	79697
			0.0476	0.1021	0.0916	0.0962	0.0766	0.0553	0.1117	0.1209	0.1090	0.0938	0.0789	0.0865				
		5	0.6050	-0.0010	0.6049	0.5057	0.7015	0.8919	0.2985	0.4943	0.5992	0.3957	0.5009	0.4996	22.00	0.00	0.00	48922
			0.0367	0.0734	0.0666	0.0757	0.0594	0.0439	0.0845	0.0897	0.0819	0.0683	0.0592	0.0639				
		7	0.6042	0.0012	0.6031	0.5036	0.6999	0.8932	0.2998	0.4954	0.5985	0.3980	0.5004	0.4987	10.00	0.00	0.00	37311
			0.0333	0.0652	0.0592	0.0687	0.0536	0.0401	0.0765	0.0805	0.0738	0.0606	0.0533	0.0572				
Alt. Mod. Asym.	PLS	2	0.4278	0.0845	0.3608	0.5773	0.7353	0.8378	0.2968	0.5676	0.6062	0.4600	0.5174	0.5164	0.00	0.00	0.00	0
			0.0367	0.0481	0.0436	0.0630	0.0406	0.0258	0.0958	0.0883	0.0849	0.0721	0.0688	0.0699				
		3	0.5233	0.0529	0.4480	0.6211	0.7899	0.8751	0.3008	0.5281	0.6013	0.4291	0.5054	0.5082	0.00	0.00	0.00	0
			0.0322	0.0477	0.0427	0.0473	0.0264	0.0144	0.0766	0.0747	0.0703	0.0582	0.0540	0.0558				
		5	0.5520	0.0413	0.4758	0.6394	0.7983	0.8812	0.2969	0.5253	0.5971	0.4177	0.5068	0.5020	0.00	0.00	0.00	0
			0.0306	0.0478	0.0422	0.0433	0.0231	0.0123	0.0719	0.0706	0.0666	0.0541	0.0499	0.0519				
		7	0.5716	0.0279	0.4984	0.6491	0.8109	0.8902	0.2976	0.5145	0.5977	0.4107	0.5036	0.5014	0.00	0.00	0.00	0
			0.0298	0.0479	0.0424	0.0411	0.0214	0.0109	0.0693	0.0692	0.0649	0.0522	0.0480	0.0500				
	OrdPLS	2	0.6109	-0.0048	0.5493	0.6634	0.8309	0.9030	0.2952	0.4939	0.5978	0.3964	0.5001	0.4970	0.00	0.00	0.00	0
			0.0494	0.0974	0.0866	0.0627	0.0358	0.0205	0.1120	0.1265	0.1125	0.0993	0.0811	0.0909				
		3	0.6063	0.0019	0.5407	0.6637	0.8314	0.9044	0.3003	0.4948	0.5977	0.3954	0.5016	0.4990	0.00	0.00	0.00	0
			0.0359	0.0646	0.0574	0.0473	0.0251	0.0130	0.0818	0.0882	0.0801	0.0675	0.0578	0.0630				
		5	0.6053	0.0027	0.5395	0.6636	0.8321	0.9039	0.2984	0.4969	0.5981	0.3981	0.4995	0.4988	0.00	0.00	0.00	0
			0.0323	0.0569	0.0505	0.0430	0.0221	0.0113	0.0741	0.0788	0.0723	0.0591	0.0519	0.0556				
		7	0.6040	0.0024	0.5388	0.6644	0.8318	0.9042	0.2987	0.4970	0.5982	0.3980	0.4997	0.4995	0.00	0.00	0.00	0
			0.0304	0.0527	0.0466	0.0404	0.0205	0.0101	0.0699	0.0730	0.0672	0.0544	0.0483	0.0514				
	PLSc	2	0.4278	0.1082	0.4623	0.3878	0.5498	0.7016	0.2968	0.5676	0.6062	0.4600	0.5174	0.5164	0.00	0.00	0.00	1024
			0.0367	0.0617	0.0575	0.0718	0.0610	0.0595	0.0958	0.0884	0.0850	0.0722	0.0687	0.0699				
		3	0.5233	0.0624	0.5270	0.4521	0.6310	0.8131	0.3008	0.5281	0.6013	0.4291	0.5054	0.5082	0.00	0.00	0.00	2971
			0.0322	0.0563	0.0514	0.0618	0.0497	0.0452	0.0766	0.0747	0.0704	0.0582	0.0539	0.0558				
		5	0.5520	0.0479	0.5523	0.4654	0.6530	0.8308	0.2969	0.5253	0.5971	0.4177	0.5068	0.5020	0.00	0.00	0.00	2996
			0.0306	0.0555	0.0499	0.0583	0.0459	0.0410	0.0720	0.0707	0.0666	0.0540	0.0500	0.0518				
		7	0.5716	0.0319	0.5695	0.4799	0.6697	0.8562	0.2976	0.5145	0.5977	0.4107	0.5036	0.5014	0.00	0.00	0.00	6490
			0.0298	0.0548	0.0492	0.0567	0.0441	0.0384	0.0693	0.0691	0.0648	0.0523	0.0480	0.0501				
	OrdPLSc	2	0.6115	-0.0061	0.6165	0.5052	0.6971	0.8828	0.2954	0.4943	0.5978	0.3961	0.5002	0.4962	75.00	0.00	0.00	145346
			0.0497	0.1104	0.0995	0.0924	0.0775	0.0642	0.1125	0.1270	0.1131	0.0995	0.0816	0.0912				
		3	0.6065	0.0026	0.6027	0.5062	0.6997	0.8934	0.3000	0.4948	0.5980	0.3950	0.5020	0.4991	17.00	0.00	0.00	51479
			0.0359	0.0719	0.0659	0.0742	0.0597	0.0441	0.0818	0.0883	0.0801	0.0675	0.0579	0.0629				
		5	0.6053	0.0029	0.6026	0.5023	0.6991	0.8939	0.2986	0.4967	0.5983	0.3981	0.4994	0.4990	7.00	0.00	0.00	36472
			0.0324	0.0635	0.0579	0.0670	0.0529	0.0401	0.0741	0.0788	0.0722	0.0590	0.0518	0.0556				
		7	0.6041	0.0028	0.6017	0.5038	0.6987	0.8943	0.2993	0.4972	0.5979	0.3981	0.4994	0.4996	8.00	0.00	0.00	24188
			0.0303	0.0588	0.0532	0.0618	0.0481	0.0371	0.0698	0.0727	0.0672	0.0544	0.0483	0.0513				

Table 6: Results for the model with two composites and one common factor

Threshold dist.	Estimator	# cat	γ_1=0.6	$\gamma_2 = 0.0$	$\beta = 0.6$	$\lambda_{y_4} = 0.5$	$\lambda_{y_5} = 0.7$	$\lambda_{y_6} = 0.9$	$w_{x_1} = 0.3$	$w_{x_2} = 0.5$	$w_{x_3} = 0.6$	$w_{y_{11}} = 0.4$	$w_{y_{12}} = 0.5$	$w_{y_{13}} = 0.5$	Load.	CompCor	notc	Boot
Alt. Ext. Asym.	PLS	2	0.3240	0.1059	0.2691	0.5489	0.6523	0.7686	0.2933	0.6288	0.5983	0.4696	0.5626	0.5101	0.00	0.00	0.00	0
			0.0421	0.0513	0.0457	0.0931	0.0847	0.0610	0.1274	0.1164	0.1147	0.0978	0.1028	0.0956				
		3	0.4224	0.0964	0.3494	0.5929	0.7024	0.8145	0.2929	0.5964	0.5958	0.4493	0.5421	0.5047	0.00	0.00	0.00	0
			0.0365	0.0482	0.0425	0.0627	0.0464	0.0306	0.0952	0.0876	0.0855	0.0718	0.0711	0.0697				
		5	0.5047	0.0674	0.4251	0.6233	0.7602	0.8580	0.2929	0.5548	0.5961	0.4285	0.5227	0.5015	0.00	0.00	0.00	0
			0.0328	0.0479	0.0422	0.0493	0.0295	0.0169	0.0791	0.0753	0.0721	0.0598	0.0562	0.0577				
		7	0.5338	0.0480	0.4601	0.6342	0.7834	0.8737	0.2893	0.5400	0.5956	0.4232	0.5143	0.4994	0.00	0.00	0.00	0
			0.0314	0.0479	0.0420	0.0455	0.0251	0.0135	0.0749	0.0725	0.0686	0.0562	0.0521	0.0540				
	OrdPLS	2	0.6554	-0.1850	0.7143	0.6479	0.8649	0.9195	0.2799	0.4442	0.5991	0.4026	0.4591	0.4896	0.00	0.00	1.00	0
			0.1258	0.8609	0.8002	0.1732	0.1485	0.1356	0.3040	0.5921	0.5213	0.3042	0.2826	0.2511				
		3	0.6130	-0.0055	0.5502	0.6652	0.8323	0.9040	0.2946	0.4933	0.5982	0.3978	0.4943	0.5005	0.00	0.00	0.00	0
			0.0502	0.1009	0.0899	0.0618	0.0378	0.0220	0.1135	0.1329	0.1172	0.1004	0.0836	0.0910				
		5	0.6043	-0.0024	0.5444	0.6624	0.8328	0.9049	0.2918	0.4966	0.5991	0.4013	0.4968	0.4994	0.00	0.00	0.00	0
			0.0370	0.0667	0.0591	0.0483	0.0259	0.0136	0.0852	0.0930	0.0838	0.0692	0.0600	0.0643				
		7	0.6052	0.0009	0.5403	0.6646	0.8315	0.9040	0.2983	0.4971	0.5976	0.3976	0.4988	0.5003	0.00	0.00	0.00	0
			0.0333	0.0589	0.0522	0.0439	0.0230	0.0118	0.0764	0.0817	0.0747	0.0610	0.0537	0.0571				
	PLSc	2	0.3240	0.1653	0.4212	0.2931	0.4302	0.5075	0.2933	0.6288	0.5983	0.4696	0.5626	0.5101	0.00	0.00	0.00	1432
			0.0421	0.0806	0.0759	0.0837	0.0742	0.0810	0.1275	0.1165	0.1146	0.0979	0.1027	0.0956				
		3	0.4207	0.1240	0.4749	0.3616	0.5322	0.6370	0.2885	0.5964	0.5948	0.4558	0.5405	0.5004	0.00	0.00	0.00	253
			0.0365	0.0646	0.0588	0.0699	0.0587	0.0593	0.0956	0.0881	0.0859	0.0716	0.0709	0.0698				
		5	0.5047	0.0818	0.5162	0.4285	0.6088	0.7637	0.2929	0.5548	0.5961	0.4285	0.5227	0.5015	0.00	0.00	0.00	1104
			0.0328	0.0582	0.0525	0.0625	0.0508	0.0475	0.0792	0.0753	0.0720	0.0598	0.0561	0.0576				
		7	0.5338	0.0564	0.5419	0.4492	0.6401	0.8074	0.2893	0.5400	0.5956	0.4232	0.5143	0.4994	0.00	0.00	0.00	1769
			0.0314	0.0565	0.0506	0.0600	0.0475	0.0431	0.0749	0.0724	0.0686	0.0562	0.0521	0.0540				
	OrdPLSc	2	0.6505	-0.1900	0.7851	0.5623	0.7094	0.8522	0.2817	0.4362	0.6057	0.3909	0.4813	0.4914	681.00	35.00	2.00	1030941
			0.1300	0.8872	0.8251	0.2236	0.1828	0.1594	0.3089	0.5995	0.5311	0.3035	0.2797	0.2499				
		3	0.6123	-0.0059	0.6102	0.5220	0.7100	0.8861	0.2964	0.4913	0.5994	0.3966	0.4963	0.4998	90.00	0.00	0.00	111322
			0.0502	0.1105	0.1015	0.0979	0.0835	0.0598	0.1138	0.1330	0.1170	0.1002	0.0832	0.0908				
		5	0.6040	-0.0021	0.6056	0.5064	0.7023	0.8946	0.2927	0.4953	0.5999	0.4007	0.4973	0.4993	28.00	0.00	0.00	64260
			0.0370	0.0740	0.0678	0.0765	0.0626	0.0453	0.0851	0.0928	0.0836	0.0693	0.0601	0.0644				
		7	0.6051	0.0008	0.6029	0.5046	0.6994	0.8938	0.2985	0.4975	0.5973	0.3980	0.4987	0.5000	14.00	0.00	0.00	42613
			0.0333	0.0655	0.0598	0.0693	0.0550	0.0412	0.0764	0.0817	0.0748	0.0608	0.0536	0.0571				
